# Characterization of the *Escherichia coli* XPD/Rad3 iron-sulfur helicase YoaA in complex with the DNA polymerase III clamp loader subunit chi (χ)

**DOI:** 10.1016/j.jbc.2022.102786

**Published:** 2022-12-09

**Authors:** Savannah J. Weeks-Pollenz, Yasmin Ali, Leslie A. Morris, Vincent A. Sutera, Elizabeth E. Dudenhausen, Margaret Hibnick, Susan T. Lovett, Linda B. Bloom

**Affiliations:** 1 Department of Biochemistry and Molecular Biology, University of Florida, Gainesville, Florida, USA; 2 Department of Biology and Rosenstiel Basic Medical Sciences Research Center, Brandeis University, Waltham, Massachusetts, USA

**Keywords:** YoaA, χ, DNA helicase, DNA repair, DNA replication, enzyme kinetics, protein purification, AZT, azidothymidine, MMS, methyl methanesulfonate, SEC, size-exclusion chromatography, SSB, ssDNA-binding protein

## Abstract

*Escherichia coli* YoaA aids in the resolution of DNA damage that halts DNA synthesis *in vivo* in conjunction with χ, an accessory subunit of DNA polymerase III. YoaA and χ form a discrete complex separate from the DNA polymerase III holoenzyme, but little is known about how YoaA and χ work together to help the replication fork overcome damage. Although YoaA is predicted to be an iron-sulfur helicase in the XPD/Rad3 helicase family based on sequence analysis, the biochemical activities of YoaA have not been described. Here, we characterize YoaA and show that purified YoaA contains iron. YoaA and χ form a complex that is stable through three chromatographic steps, including gel filtration chromatography. When overexpressed in the absence of χ, YoaA is mostly insoluble. In addition, we show the YoaA-χ complex has DNA-dependent ATPase activity. Our measurement of the YoaA-χ helicase activity illustrates for the first time YoaA-χ translocates on ssDNA in the 5ˈ to 3ˈ direction and requires a 5ˈ single-stranded overhang, or ssDNA gap, for DNA/DNA unwinding. Furthermore, YoaA-χ preferentially unwinds forked duplex DNA that contains both 3ˈ and 5ˈ single-stranded overhangs *versus* duplex DNA with only a 5ˈ overhang. Finally, we demonstrate YoaA-χ can unwind damaged DNA that contains an abasic site or damage on 3ˈ ends that stall replication extension. These results are the first biochemical evidence demonstrating YoaA is a *bona fide* iron-sulfur helicase, and we further propose the physiologically relevant form of the helicase is YoaA-χ.

The *Escherichia coli* gene *yoaA* encodes a putative XPD/Rad3-like iron-sulfur helicase. XPD/Rad3-like helicases are Super Family 2 helicases that use ATP hydrolysis for energy to translocate in the 5ˈ to 3ˈ direction on DNA to unwind DNA/DNA and DNA/RNA complexes (reviewed in (1)). XPD/Rad3-like helicases contain four domains—two helicase domains, an iron-sulfur cluster (Fe-S) domain, and arch domain. The Fe-S cluster and arch domains are unique to XPD/Rad3-like helicases and are inserted into helicase domain 1. The Fe-S cluster is necessary for helicase activity and is believed to have multiple roles including aiding in the physical separation of ds DNA to form ssDNA and sensing DNA damage (2, 3, 4, 5, 6, 7). The Fe-S cluster has also been proposed to have a role in sensing redox potential, and the reversible reduction of the Fe-S cluster turns off helicase activity of the YoaA paralog, DinG (4, 8). All domains of life contain XPD/Rad3-like helicases, and the four human helicases (FANCJ, RTEL1, CHLR1/DDX11, and XPD) are crucial for maintaining genomic stability (1, 9, 10, 11). Mutations in any one of the four human genes is associated with genomic instability and disease (12, 13, 14, 15). *E. coli* has two XPD/Rad3-like helicases, DinG, a damage-inducible helicase, and the putative helicase, YoaA. At the protein sequence level, *yoaA* contains seven conserved XPD/Rad3-like helicase motifs including a Walker A Box (nucleoside triphosphate binding), Walker B Box (nucleoside triphosphate hydrolysis), and four cysteines believed to coordinate the iron in the Fe-S cluster (16). Mutating one of these three conserved motifs causes *yoaA* to no longer confer azidothymidine (AZT) tolerance, implying these are necessary for the biological activity of YoaA (16).

Genetic studies showed YoaA has a physical and functional interaction with the DNA polymerase III clamp loader accessory subunit, χ, and together, they contribute to DNA damage tolerance (16, 17, 18, 19). Genetic screens in *E. coli* identified *yoaA* and *holC* (χ) to be critical for rescuing stalled DNA replication forks when AZT was used as a chemical tool to halt DNA synthesis (16). The genes *yoaA* and *holC* are also important for repairing methyl methanesulfunoate (MMS) lesions (18). MMS is an alkylating agent that generates DNA adducts that can lead to replication stalling (20). In addition, *yoaA* expression is regulated by the SOS response in bacteria and is induced in the presence of DNA damage (21, 22). To date, genetic studies have investigated functions of *yoaA* and *holC* in *E. coli* but YoaA has not yet been characterized biochemically (16, 18, 19, 21, 22, 23).

The observation that *holC* or χ was required along with *yoaA* for DNA damage tolerance implicated DNA polymerase III holoenzyme (pol III HE) or the clamp loader unit of the holoenzyme in this pathway. However, χ was found to coelute with His-tagged YoaA from a Ni^2+^-sepharose column, whereas the ψ subunit of pol III HE flowed through the column and was not retained with His-tagged YoaA and χ (19). Given that the only binding site for χ in the holoenzyme is on the ψ subunit, this result demonstrated that χ binds either YoaA or the pol III HE, but not both at the same time (24, 25). Thus, this work suggests that χ may function as a subunit of a YoaA helicase.

This work characterizes the fundamental biochemical activities of YoaA and its interactions with χ. Chi enhances the solubility of YoaA and a *bona fide* YoaA-χ complex forms when the proteins are overexpressed *in vivo*. YoaA-χ has the canonical XPD/Rad3-like helicase enzymatic activities including DNA-dependent ATPase activity, DNA/DNA unwinding activity, and translocates with a 5ˈ to 3ˈ polarity. This work is the first biochemical evidence of YoaA as an XPD/Rad3-like helicase, and given the tight binding of χ with YoaA, we propose that the physiologically relevant form of the helicase is YoaA-χ.

## Results

### The clamp loader accessory subunit of DNA polymerase III, χ, increases the solubility of YoaA

The YoaA protein used in this work contains an N-terminal 6× histidine fusion tag (His-YoaA). Because YoaA contains four cysteines (C108, C168, C173, and C179) that are expected to coordinate four iron atoms to form a Fe-S cluster, media for expression of His-YoaA was supplemented with iron (II) sulfate (0.1 mg/ml) and ammonium ferric citrate (0.1 mg/ml) to supply additional iron for the formation of the Fe-S cluster. Purified fractions of His-YoaA had a yellow tint, indicative of an iron-containing protein. The presence of iron was verified using a colorimetric assay (see Experimental procedures).

**Figure 1:**
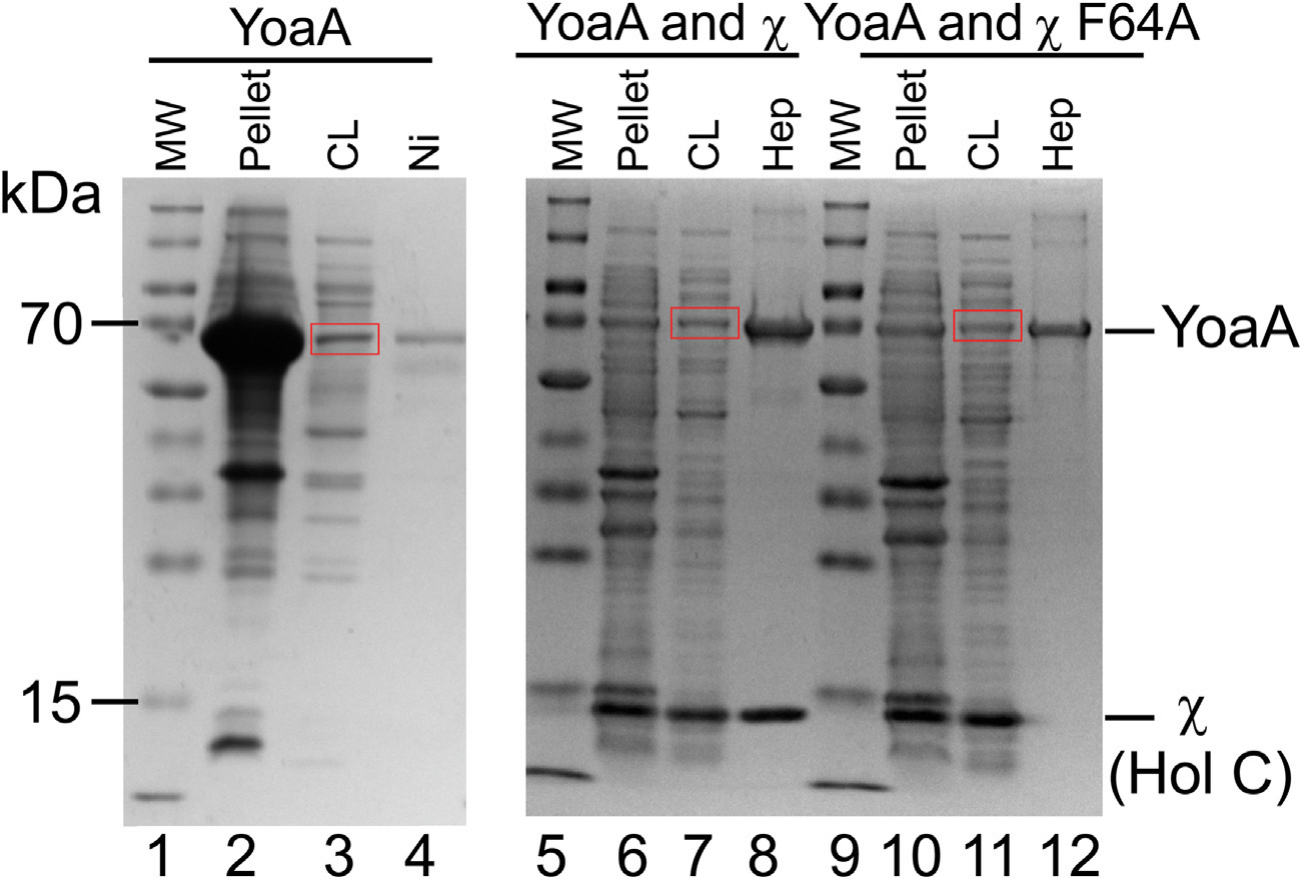
**Chi increases the solubility of YoaA.** SDS-PAGE gels, stained with Coomassie Brilliant Blue, of three different overexpression conditions: *left panel* is 6X-His-tagged YoaA, *middle panel* is χ and 6X-His-tagged YoaA, and *right panel* is χ F64A and 6X-His-tagged YoaA. Lanes are numbered and labeled as follows: molecular weight markers (MW), insoluble material from clarified lysate (pellet), supernatant from clarified lysate (CL), proteins eluting from Ni^2+^ column with imidazole (Ni), and proteins eluting from a heparin column (Hep). Expected size of 6X-His-YoaA is 72.6 kDa and 16.6 kDa for χ. *Red boxes* outline His-YoaA in the soluble fractions. Equal volumes were loaded for all lanes.

Overexpression of His-YoaA (72.6 kDa) alone from a pCOLADuet vector in *E. coli* BL21(DE3) cells produced a large fraction of insoluble protein after cell lysis (Fig. 1, lane 2, band at about 70 kDa). A small fraction of soluble YoaA was present in the soluble fraction that was enriched after purification by metal ion affinity chromatography (Ni^2+^ sepharose) (Fig. 1, lanes 3 and 4). When expressed alone, YoaA yields are low making it difficult to purify enough for biochemical assays. Overexpressing His-YoaA with χ increased the solubility of YoaA (Fig. 1, lane 7). His-YoaA was still present in the insoluble fraction (Fig. 1, lane 6), but the percentage of YoaA compared to other proteins in the insoluble fraction was reduced compared to cells overexpressing His-YoaA alone (Fig. 1, lanes 2 and 6). Chi (16.6 kDa) copurified with YoaA on Ni^2+^ affinity and heparin columns (Fig. 1, lane 8).

Mutation of Phe-64 to Ala in χ (χ F64A) weakens binding of χ to YoaA (19). The χ F64A mutant is soluble and expressed at high levels. His-YoaA and the χ F64A mutant were coexpressed to determine if higher yields of soluble His-YoaA could be obtained and His-YoaA could be purified from χ F64A. Expressing His-YoaA with the χ F64A mutant increased the solubility of YoaA, but the interaction between χ F64A and YoaA was too weak for the two proteins to copurify (Fig. 1, lane 12). Chi F64A was present in the soluble fraction indicating χ F64A was overexpressed but did not copurify with His-YoaA (Fig. 1, lanes 11 and 12).

### YoaA and χ form a complex

**Figure 2:**
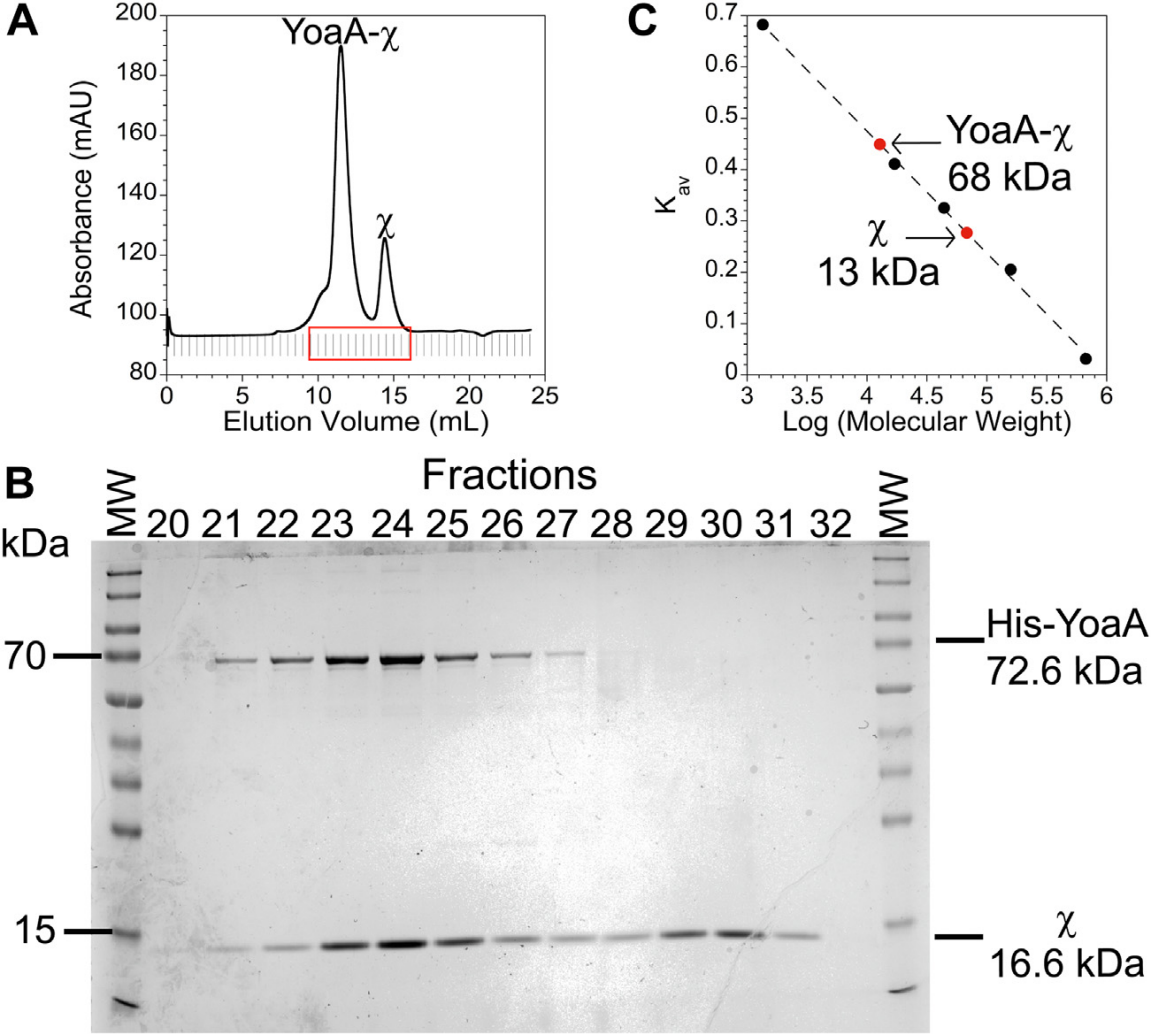
**YoaA is a monomer that forms a complex with χ.***A*, the UV absorbance of column fractions eluted from a Superose 12 10/300 gel filtration column is shown. The larger peak corresponds to YoaA-χ and the smaller peak to χ only. *Vertical lines* represent fractions and *red box* outlines fractions in panel (*B*). *B*, SDS-PAGE analysis of fractions 20 through 32 eluted from SEC column is shown. MW denotes molecular weight ladder. Expected size of His-YoaA is 72.6 kDa and is 16.6 kDa for χ. *C*, the SEC column was calibrated with protein standards by graphing K_av_*versus* log (molecular weight) (*black filled circles*). The standard curve was fit to a line (R^2^ of 0.998) to calculate molecular weights of YoaA-χ and χ (*red filled circles*). One representative experiment is shown, and average size calculated from two experiments for YoaA-χ and χ on the Superose 12 column is 68 and 13 kDa, respectively. SEC, size-exclusion chromatography.

His-YoaA copurifies with χ on two successive columns, Ni^2+^ affinity and heparin column (Fig. 1). To verify that His-YoaA and χ form a protein complex and to determine the stoichiometry of the proteins, His-YoaA and χ were coexpressed, purified by Ni^2+^ affinity and heparin chromatography, and subjected to size-exclusion chromatography (SEC) on a Superose 12 column (Fig. 2*A*). Two peaks eluted from SEC between fractions 20 through 32 and these fractions were analyzed by SDS-PAGE to identify the proteins corresponding to the peaks (Fig. 2, *A* and *B*). Fractions 23-25 contained two strong bands at approximately 70 kDa and 15 kDa, corresponding to the expected sized of His-YoaA and χ, whereas fractions 29-31 contained one strong band at approximately 15 kDa, corresponding to χ. The presence of the small χ protein in the early eluting fractions with the larger His-YoaA confirms the two proteins form a stable complex. Using protein standards ranging in size from 1.4 kDa to 670 kDa, the molecular weights of the His-YoaA–χ complex and χ were calculated from an average of two experiments to be 68.3 kDa and 13.1 kDa, respectively. The calculated molecular weight of the observed YoaA-χ complex (68.3 kDa) is smaller than the expected size of a 1:1 His-YoaA:χ complex (89.2 kDa). His-YoaA and χ were coexpressed and purified as above and were also analyzed using a different SEC column, a Superdex 200 Increase column. As on the Superose 12 column, His-YoaA and χ coeluted in the same peak. Calibration of the Superdex 200 column with standards gave an apparent molecular weight of 98.0 kDa for His-YoaA-χ (Fig. S1). Due to the insolubility of YoaA without χ and the observation that YoaA and χ form a tight complex, we focused on characterization of the enzymatic features of YoaA-χ.

### YoaA-χ has DNA-dependent ATPase activity

**Figure 3:**
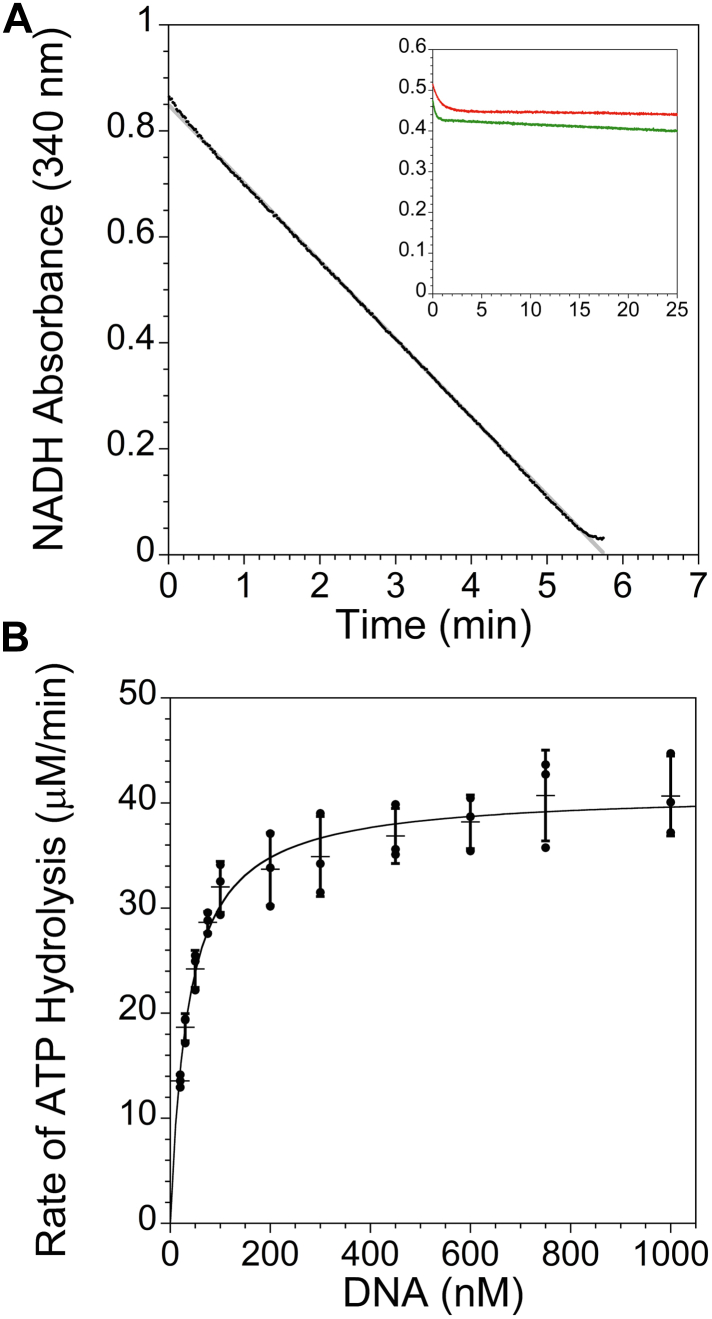
**YoaA-χ has DNA-dependent ATPase activity.***A*, the NADH absorbance at 340 nm as a function of time for an ATPase-coupled assay of YoaA-χ (25 nM) on a 60-nt ssDNA (1 μM, Table S2, substrate S1). The decrease in NADH absorbance was fit to a line (R^2^ of 0.999, *gray*) to obtain a rate from the slope (−0.147 min^−1^). The inset shows ATPase reactions for YoaA K51A-χ (25 nM, *green*) with a 60-nt ssDNA (1 μM, S1) compared to wt YoaA-χ (25 nM) with no DNA (*red*). Each experimental condition has been performed in triplicate. *B*, rates of ATP hydrolysis by YoaA-χ (25 nM) on 60-nt ssDNA (S1) as a function of DNA concentration. DNA concentration ranged from 20 nM to 1 μM. Data were fit to a Michaelis-Menten–like equation, $y=\frac{V_{\max}x}{K_{0.5}+x}$. The average *K*_*0.5*_ for three independent experiments for DNA is 35.7 ± 2.9 nM. Data for three experiments (*filled circles*) along with averages (*horizontal lines*) and SDs (error bars) are shown.

Helicases typically use ATP as the energy source for translocating along ssDNA, and mutation of the lysine in the Walker A motif of YoaA showed that YoaA needs to bind and hydrolyze ATP for AZT tolerance *in vivo* (16). Therefore, ATPase activity of YoaA was measured *in vitro* to determine whether ATPase is potentially coupled to translocation on DNA. An enzyme-coupled assay that links the conversion of ADP product to the oxidation of NADH was used to measure ATPase activity. ATP hydrolysis was not detected for YoaA-χ in the absence of DNA (Fig. 3*A* inset, red). However, when a 60-nucleotide (nt) ssDNA substrate (Table S2, substrate S1) was added, YoaA-χ hydrolyzed ATP showing that ATPase activity of YoaA-χ is dependent on DNA (Fig. 3*A*, black). The rate of ATPase activity of YoaA-χ was also dependent on the concentration of ssDNA, and the concentration of DNA required to achieve half-maximal ATPase activity, *K*_*0.5*_, is 35.7 ± 2.9 nM (Fig. 3*B*).

Since ATPases require Mg^2+^ for coordination and catalysis, the optimal MgCl_2_ concentration for the ATPase activity of YoaA-χ for the same substrate (S1) was determined. The rate of ATP hydrolysis by YoaA-χ was dependent on MgCl_2_ concentration and 2.5 and 5 mM MgCl_2_ yielded the fastest rates of ATP hydrolysis (Fig. S2). YoaA-χ had no measurable ATPase activity in the absence of MgCl_2_ (data not shown).

### Characterization of the helicase activity of YoaA-χ

**Figure 4:**
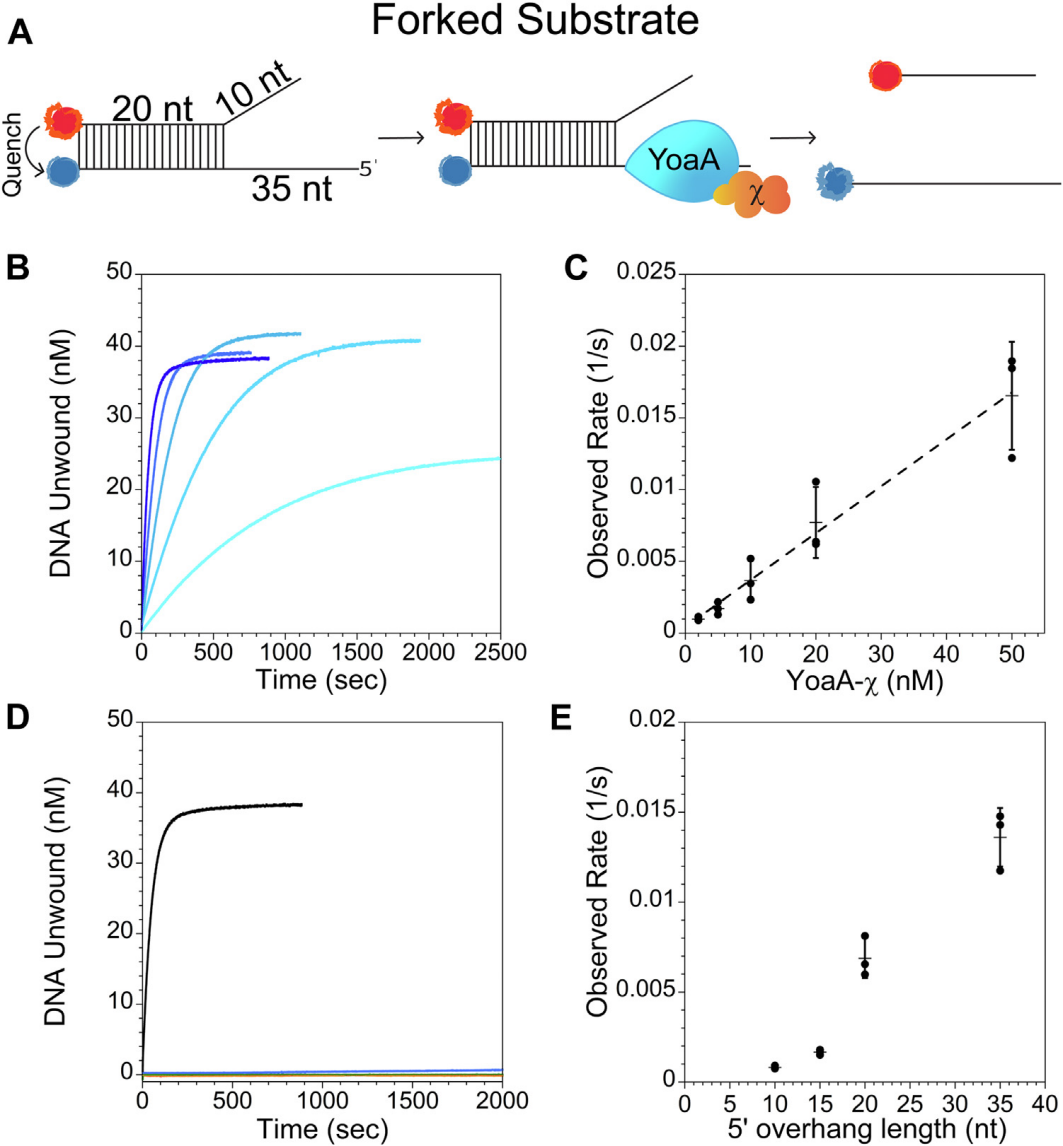
**YoaA-χ requires ATPase activity to unwind DNA/DNA-forked duplexes.***A*, schematic of FRET-based helicase assay where the presence of Cy5 (*red*) near Cy3 (*blue*) quenches the fluorescence of Cy3. Cy3 fluorescence will increase when the DNA strands are separated by YoaA-χ. *B*, representative time courses for forked DNA/DNA duplex (50 nM, Table S3, substrate F1) unwound by increasing concentrations of YoaA-χ (2 nM, 5 nM, 10 nM, 20 nM, and 50 nM) are shown with darkness in *blue* increasing the concentration. The increase in fluorescence of Cy3 was converted to DNA concentration as described in the Experimental procedures. *C*, the observed rates of DNA molecules unwound is plotted *versus* YoaA-χ concentration and fit to a line. The slope from three experiments (indicated by *filled circles*) is 33 × ^−5^ nM^−1^s^−1^ with an R^2^ of 0.996. Average values are shown by *horizontal lines* and error bars represent SD. *D*, representative time courses for forked DNA (50 nM, F1) unwound by wt YoaA-χ (50 nM) with ATP (*black*), without ATP (*red*), with ATPγS (*blue*), or with AMP-PNP (*orange*), and YoaA K51A-χ (50 nM) with ATP (*green*). Each time course was performed three times. *E*, the observed rates of DNA unwound by YoaA-χ (50 nM) for forked DNA with either a 10-, 15-, 20-, or 35-nt 5ˈ ss overhang length (50 nM, F1-F4). Individual values for three experiments (*filled circles*) with average values (*horizontal lines*) and error bars representing SD are shown.

A FRET-based assay was utilized to measure the helicase characteristics of YoaA-χ. A 30-nt oligonucleotide labeled with Cy5 on the 5ˈend was annealed to a 55-nt oligonucleotide labeled with Cy3 on the 3ˈ end to generate a forked DNA substrate where the blunt end of the 20-nt duplex is labeled with a fluorescent donor (Cy3) and acceptor (Cy5) (Table S3, substrate F1) (Fig. 4*A*). The DNA substrate is bifurcated with a 10-nt ss overhang on the Cy5-labeled strand and 35-nt ss overhang on the Cy3-labeled strand. Due to the close proximity of Cy5 to Cy3, the fluorescence of Cy3 is quenched by Cy5. When YoaA-χ unwinds the entire 20-nt of ds DNA, the two strands will separate and Cy3 fluorescence will increase (Fig. 4*A*). Helicase reactions contained 2 mM ATP and 50 nM forked DNA (F1) and were initiated by adding YoaA-χ to DNA and ATP (Fig. 4*B*). The fluorescence intensity of Cy3 was converted to amount of DNA unwound by using the Cy3 signal of ds and ssDNA without YoaA-χ as low and high signals, respectively. YoaA-χ readily unwound this forked DNA substrate (Fig. 4*B*). The observed rate of DNA molecules unwound per time increased linearly with YoaA-χ concentration (Fig. 4*C*). It has been previously established that the Fe-S cluster in helicases, such as XPD, can quench Cy3 and Cy5 (3, 26, 27). However, under our experimental conditions, YoaA-χ does not significantly quench the fluorescence of Cy3 at the two highest concentrations used (20 nM and 50 nM) for this DNA substrate (Table S2, substrate S2) (Fig. S3). Lack of Fe-S–dependent quench is most likely due to having a small population of DNA molecules bound by YoaA-χ near Cy3 at any given time in our experiments.

This FRET helicase assay is attractive because it gives a signal that can be measured directly as a function of time; however, a limitation is that any process that separates the donor and acceptor pair such as nucleolytic degradation of DNA will give a FRET signal. To verify that the FRET signal is due to helicase activity of YoaA-χ, the FRET helicase assay was complemented with a DNA gel helicase assay. A forked DNA substrate (50 nM, G1), containing a longer 5ˈ ssDNA overhang to maximize separation on a native gel, was unwound by 2 nM and 5 nM YoaA-χ in a concentration- and time-dependent manner (Fig. S4). DNA degradation by a nuclease was not detected in these gel assays nor was nuclease activity detected by a denaturing PAGE for an ssDNA substrate with a fluorescein located in the middle of the strand (data not shown). Together, these results confirm that YoaA-χ has DNA helicase activity which is responsible for the increase in fluorescence in FRET assays, and YoaA-χ is not contaminated with a nuclease.

YoaA was coexpressed with χ F64A and purified away from χ F64A to obtain YoaA in higher yields to measure the rate of ATP hydrolysis and DNA unwinding of YoaA alone. In the absence of χ, YoaA had approximately an 85% decrease in ATPase and DNA unwinding activity (performed once, data not shown). Although these preliminary results suggest χ stimulates the activity of YoaA, it is possible that YoaA is unstable in the absence of bound χ. These experiments were performed with one preparation of YoaA χF64A and further characterization needs to be performed to confirm the role χ has with YoaA in the helicase complex.

### DNA unwinding is ATP-dependent

It is the translocation of helicases along ssDNA that separates annealed DNA strands, and XPD/Rad3-like helicases require ATP hydrolysis to translocate. To determine whether YoaA-χ requires ATP binding or ATP hydrolysis for DNA unwinding, unwinding activity was measured for a mutant enzyme and using nonhydrolyzable ATP analogs. The conserved Lys residue in the Walker A motif of YoaA was mutated to Ala (YoaA K51A) to reduce ATP binding and, therefore ATP hydrolysis. YoaA K51A-χ lacked ATPase activity (Fig. 3*A* inset, green). This confirmed that this K51A mutation results in an ATPase defective enzyme. The Walker A mutant was also unable to unwind DNA under conditions where the WT enzyme does (Fig. 4*D*). Given that the K51A mutation affects ATP binding, experiments were also done to determine whether ATP hydrolysis specifically was required for DNA unwinding by utilizing hydrolysis-resistant ATP analogs, adenosine 5ˈ-(γ-thio)-triphosphate (ATPγS) and adenylyl-imidodiphosphate (AMP-PNP). Neither ATP analog supported DNA unwinding of the forked DNA substrate (F1) by wt YoaA-χ, and DNA unwinding did not occur in the absence of ATP (Fig. 4*D*). Together, these experiments show that the DNA unwinding activity of YoaA-χ is dependent on a canonical ATP-binding residue and on ATP hydrolysis.

### The rate of unwinding forked substrates increases with the length of the 5ˈ ss overhang

To determine how long the 5ˈ ssDNA overhang must be for YoaA-χ to efficiently unwind ds DNA, the rate of unwinding bifurcated DNA (50 nM) with a 10-nt 3ˈ ss overhang and either a 10-, 15-, 20-, or 35-nt 5ˈ ss overhang (Table S3, substrates F1-F4) was measured using the FRET helicase assay. Unwinding by YoaA-χ (50 nM) was barely detectable on a substrate with a 10-nt 5ˈ ss overhang (F2) under these conditions (0.8 ± 0.09 × 10^−3^ s^−1^) (Fig. 4*E*). The rate of unwinding doubled when the length of the 5ˈ overhang was increased to 15-nt (F3, 1.7 ± 0.1 × 10^−3^ s^−1^), increased eight fold with a 20-nt 5ˈ ss overhang (F4, 6.9 ± 1 × 10^−3^ s^−1^), and increased 16 fold with a 35-nt 5ˈ ss overhang (F1, 13 ± 4 × 10^−3^ s^−1^) when compared to a 10-nt 5ˈ ss overhang (Fig. 4*E*).

### YoaA-χ translocates 5ˈ to 3ˈ on DNA

**Figure 5:**
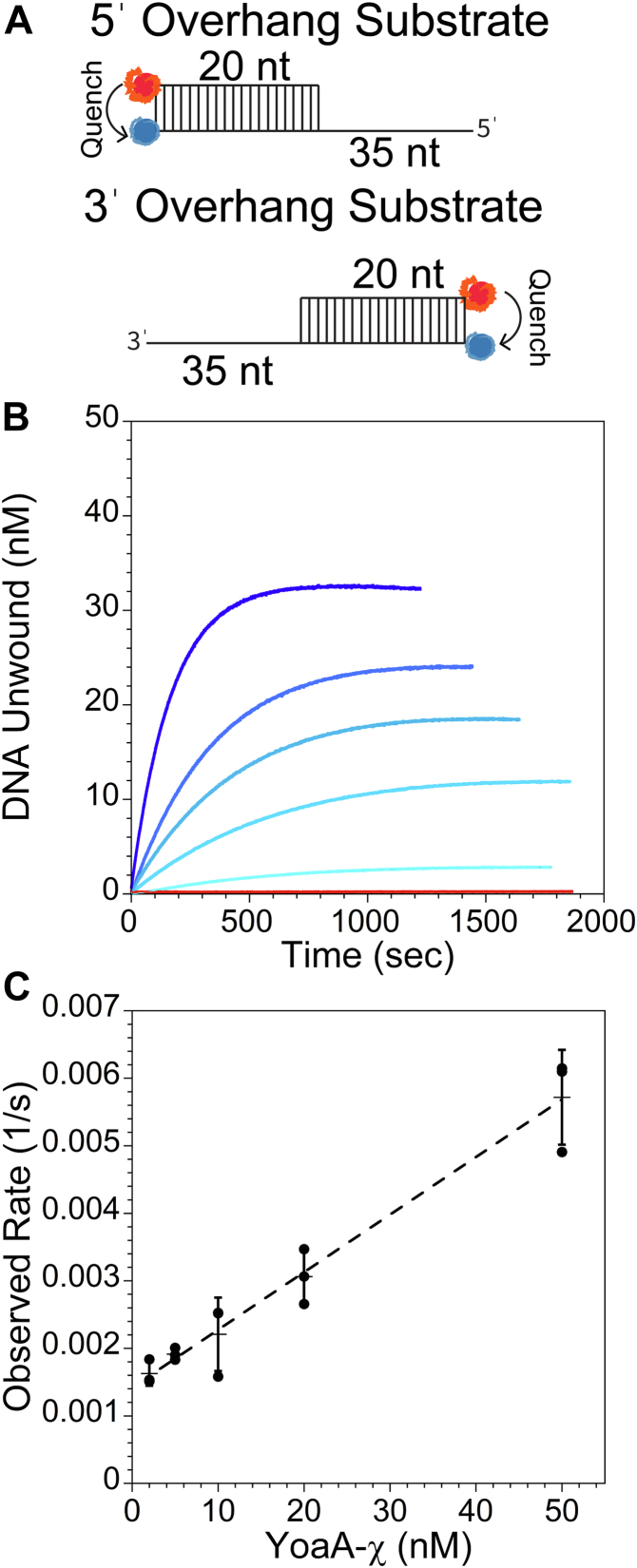
**YoaA-χ unwinds DNA/DNA-forked duplexes in the 5ˈ to 3ˈ direction.***A*, schematic of 5ˈ and 3ˈ ss overhang DNA substrates used for the FRET helicase assay in (*B* and *C*). *B*, representative time courses for DNA unwinding of 5ˈ or 3ˈ overhang DNA/DNA duplex substrate (50 nM, Table S3, O1 and O2) by YoaA-χ. Reactions with the 5ˈ ss overhang contained increasing concentrations of YoaA-χ (2 nM, 5 nM, 10 nM, 20 nM, and 50 nM) where the darkness of *blue* increases with concentration, and the reaction for the 3ˈ ss overhang (*red*) was performed at the highest YoaA-χ concentration (50 nM). *C*, the observed rate of 5ˈ overhang DNA molecules unwound per time is plotted *versus* YoaA-χ concentration and fit to a line. The slope from three experiments (*filled circles)* is 8.5 × 10^−5^ nM^−1^s^−1^ with an R^2^ of 0.999. Average values are shown by *horizontal lines* and error bars represent SD.

XPD/Rad3-like helicases translocate in the 5ˈ to 3ˈ direction on DNA, and YoaA is predicted to translocate in the same direction (28, 29, 30, 31, 32). Helicase activity of YoaA-χ was measured on two different substrates to determine the polarity of translocation. Both substrates contained a 20-nt DNA duplex with either a 5ˈ or 3ˈ 35-nt ss overhang (Table S3, substrates O1 and O2, respectively) (Fig. 5*A*). The blunt end of the duplex was labeled with the Cy3-Cy5 FRET pair to measure strand separation. Cy3 fluorescence increased in the presence of YoaA-χ for the DNA substrate with a 5ˈ ss overhang (O1) (Fig. 5*B*). At the highest concentration used (50 nM), YoaA-χ did not unwind the DNA substrate with a 3ˈ ss overhang substrate (O2) (Fig. 5*B*, red trace). Similar to the forked substrate (F1), the observed rate of unwinding the 5ˈ overhang substrate (O1) increased linearly with YoaA-χ concentration (Fig. 5*C*). Interestingly, the DNA unwinding reaction was about four times faster for the forked substrate (F1) with a slope of 33 × 10^−5^ nM^−1^s^−1^ than the reaction for the overhang substrate (O1) with a slope of 8.5 × 10^−5^ nM^−1^s^−1^ (Figs. 4*C* and 5*C*). To investigate if unwinding was faster for a bifurcated substrate because of the unpaired nucleotides, we measured the rate of unwinding for YoaA-χ on a substrate with one unpaired nucleotide on the 3ˈ end (F5). Adding one unpaired nucleotide to the 3ˈ end had little effect on the rate of unwinding compared to an overhang substrate (O1), with a rate of 6.0 ± 1 × 10^−3^ s^−1^ *versus* 4.7 ± 1 × 10^−3^ s^−1^, respectively (see Fig. 8, F5 and O1).

### YoaA-χ binding to DNA with or without a 3ˈ ss overhang

**Figure 6:**
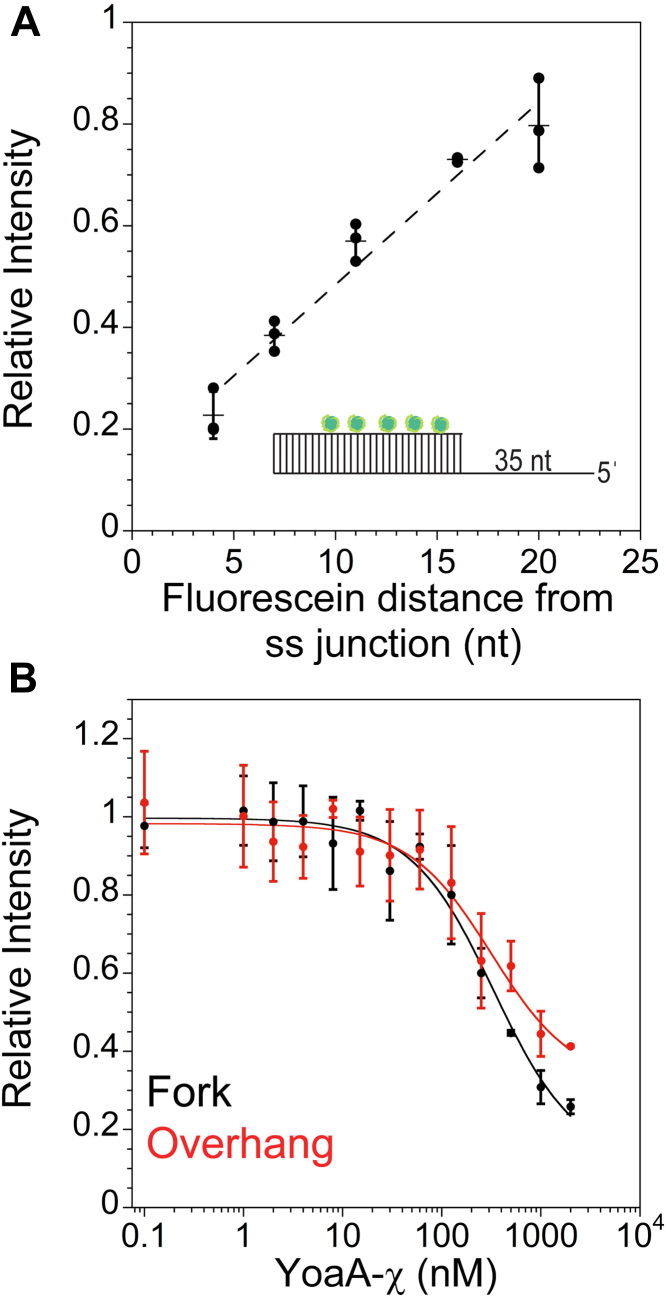
**Comparing YoaA-χ binding to bifurcated DNA *versus* 5ˈ ss overhang DNA.***A*, quench in fluorescence that occurs when 1 μM YoaA-χ is added to 50 nM DNA that is labeled with fluorescein on the duplex 4-, 7-, 11-, 16-, or 20-nt away from the ds/ss junction (Table S3, substrate B1). Data from three experiments (*filled circles*) along with averages (*horizontal lines*) and SDs (error bars) are shown. These data were fit to a line (R^2^ value of 0.968). *B*, titration of 50 nM fluorescein-labeled overhang substrate (B2, *red*) or 50 nM fluorescein-labeled forked substrate (B3, *black*) with YoaA-χ (0.1 nM – 2 μM). Data were fit with Equation 6 to calculate *K*_*d*_ values (Experimental procedures). *Filled circles* represent the average of three measurements and error bars represent SDs.

The Fe-S cluster in Rad3/XPD family helicases quenches fluorescence of fluorophores covalently bound to DNA in a distance-dependent manner (3, 26, 27). To determine whether YoaA would similarly quench fluorescence when bound to labeled DNA, a DNA substrate containing a 30-nt duplex and 35-nt 5ˈ ss overhang was labeled with fluorescein at different sites along the duplex. Five different DNA substrates with fluorescein at 4-, 7-, 11-, 16-, and 20-nt from the 3ˈ end of the ss/ds DNA junction were tested (Table S3, substrate B1). Binding reactions contained 50 nM fluorescein-labeled DNA, 1 μM YoaA-χ, and 0.5 mM ATPγS to allow YoaA-χ to bind but not unwind DNA (Fig. 6*A*). When fluorescein was 4-nt from the ss/ds junction, the largest quench of approximately 80% occurred whereas only a 20% quench occurred when fluorescein was 20-nt away from the junction. The quench in fluorescence varied relatively linearly with the position of the fluorophores along the duplex. This distance-dependent quench provides additional evidence that YoaA contains a Fe-S cluster.

We used the fluorescein quench as a method to measure DNA-binding affinity of YoaA-χ for bifurcated duplex as in Figure 4 and DNA lacking the 3ˈ ss overhang as in Figure 5 to determine whether differences in helicase activity are due to differences in binding affinity. Fluorescein-labeled DNA (50 nM, B2 and B3) was titrated with YoaA-χ (0.1 nM to 2 μM) (Fig. 6*B*). The binding affinity was so low that saturation could not be achieved within experimentally accessible concentrations of YoaA-χ. Binding data were fit to the quadratic equation assuming a 1:1 YoaA-χ:DNA–binding model to give rough estimates of *K*_*d*_ values of 328 ± 166 nM for the forked substrate and 350 ± 329 nM for the substrate with only a 5ˈ ssDNA overhang (Fig. 6*B*). These fits along with the similarity of the binding isotherms indicate that the affinity of YoaA-χ for both substrates are on the same order of magnitude. These data also explain why enzyme-dependent quenching of fluorescence was not observed in FRET helicase assays (Fig. S3). The enzyme concentration used in the FRET helicase assays was too low for a substantial fraction of DNA to be bound.

### YoaA-χ unwinds gapped DNA and 5ˈ flap forked structures

**Figure 7:**
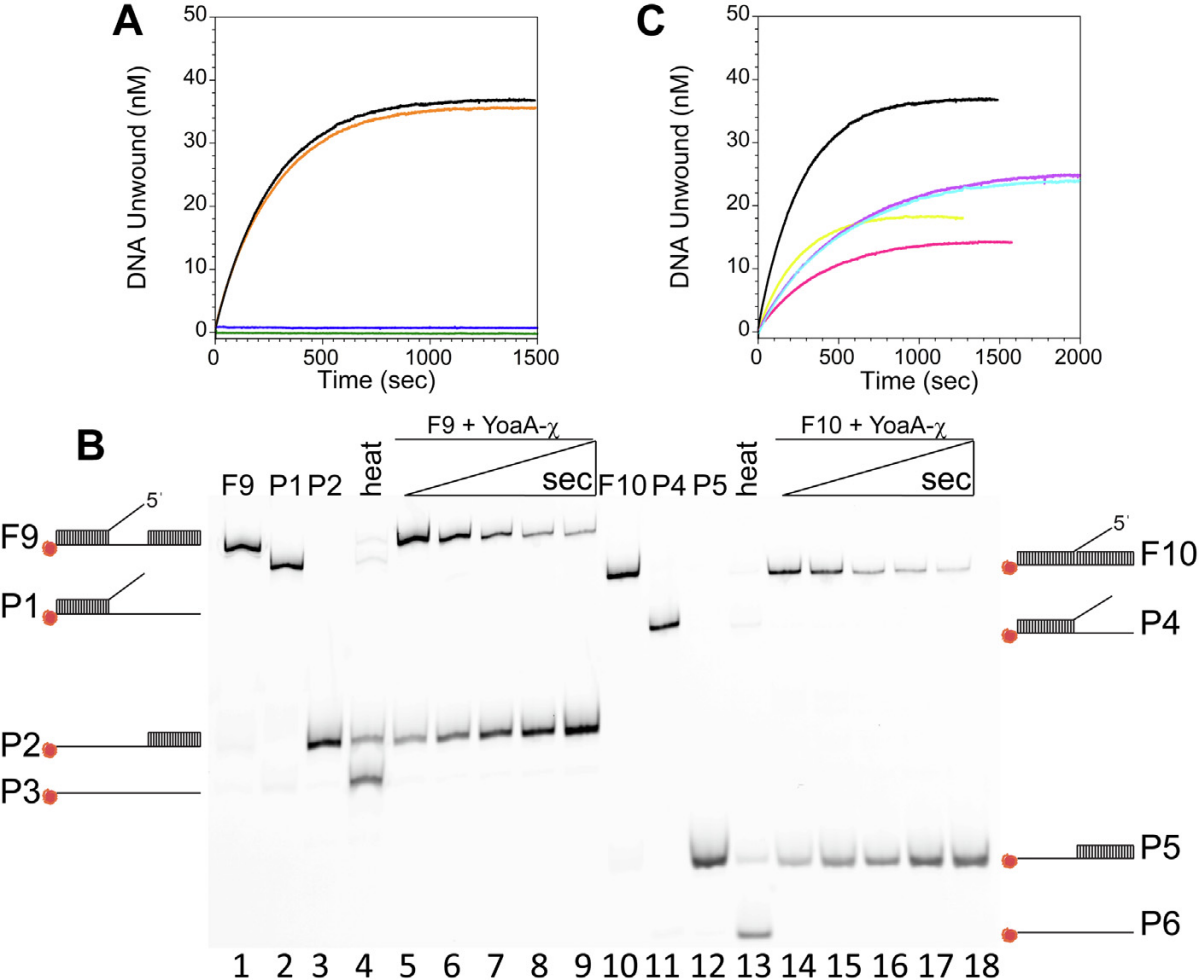
**YoaA-χ unwinds forked and damaged DNAs.***A*, representative time courses for unwinding duplex DNA with either a 20-nt gap (50 nM, Table S3, substrate F6, *orange*), a nick (50 nM, F7, *green*), or a nick with a 35-nt 3ˈ flap (50 nM, F8, *blue*) by YoaA-χ (50 nM). Duplex DNA with a 35-nt 5ˈ ss overhang (50 nM, O1) unwound by the same YoaA-χ preparation is overlaid for comparison (*black*). *B*, native gel showing 50 nM duplex DNA with a 20-nt gap (F9) or nick (F10) unwound by YoaA-χ (50 nM) at 15 s, 30 s, 60 s, 300 s, and 900 s. Lanes 1-4 (*left panel*) and lanes 10-13 (*right panel*) are DNA-only controls to serve as markers for the substrates F9 (lane 1) and F10 (lane 10) and respective, possible products P1-P3 (lanes 2–4) and P4-P6 (lanes 11–13). The substrates were heated at 95 °C in lanes 4 and 13 to generate products. *C*, representative time courses for unwinding damaged, duplex DNA (50 nM) with either AZT on the 3ˈ end of the primer (D1, *purple*), 3ˈ dideoxy C on the 3ˈ end of the primer (D2, *yellow*), or an abasic site 1-nt after the ds/ss junction on the translocating strand (D3, *light blue*) by YoaA-χ (50 nM). Also showing representative time courses for unwinding undamaged, duplex DNA (50 nM) with either a C/G bp at the ds/ss junction (O3, *pink*) or T/A bp (O1, *black*, experiment from *A*) by YoaA-χ (50 nM). Each experimental condition has been performed with three repeats for panels (*A*–*C*). AZT, azidothymidine.

**Figure 8:**
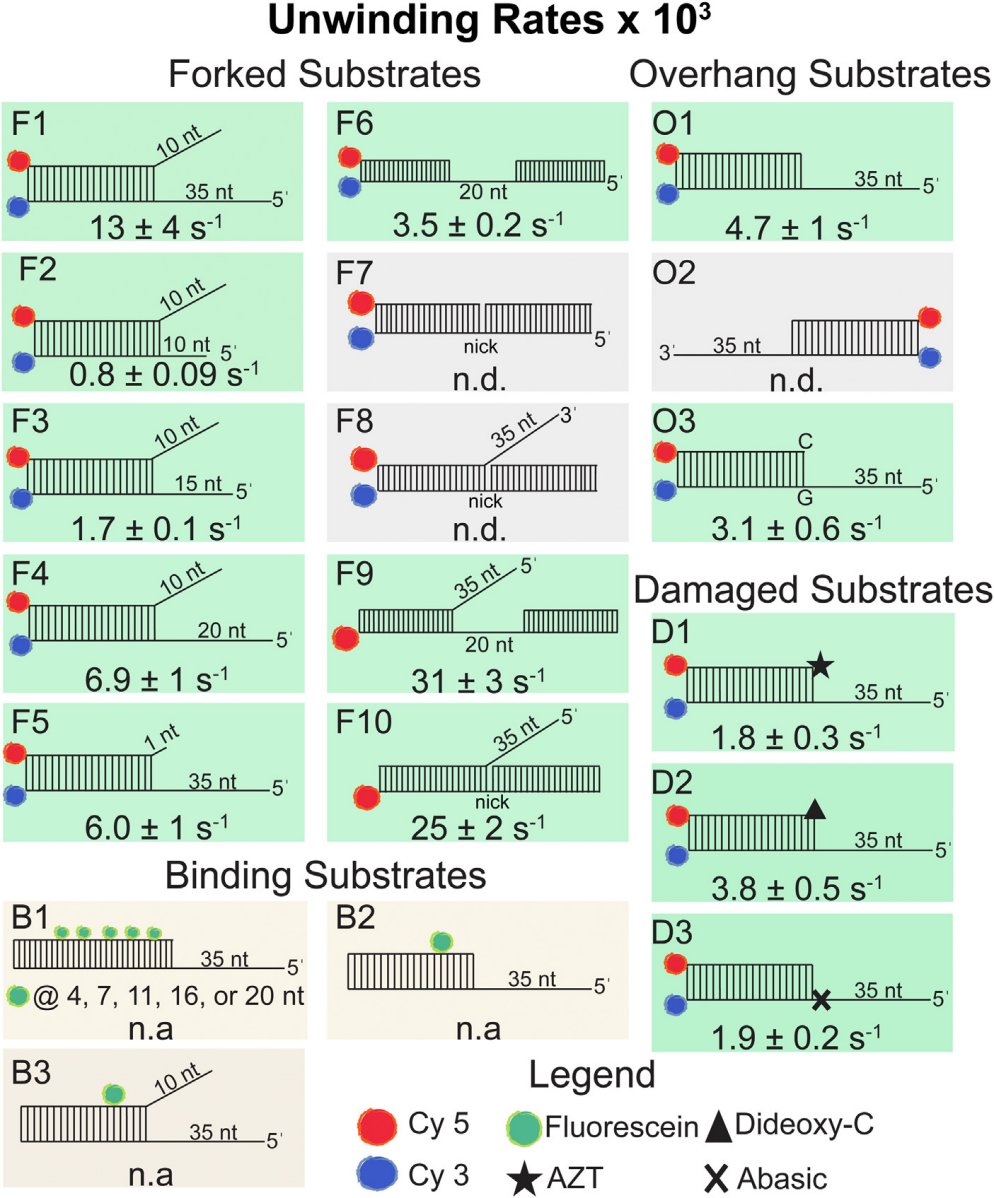
**Comparison of unwinding rates by YoaA-χ for all substrates tested.***Green boxes* are substrates unwound by YoaA-χ and *gray boxes* are substrates not unwound by YoaA-χ in reactions with 50 nM YoaA-χ, 2 mM ATP, and 50 nM DNA. The average rate of unwinding from three measurements multiplied by 10^3^ is indicated below the substrate along with the SD, or if unwinding was not detectable, n.d. is indicated. For substrate F1, the average is from nine experiments performed throughout the paper with four different preparations of YoaA-χ. For substrate O1, the average is from six experiments with four different preparations of YoaA-χ. Duplex regions of DNA substrates are 20-nt, except for substrate B1, which is 30-nt. *Yellow boxes* indicate substrates used to measure YoaA-χ binding. For B2 and B3, the fluorescein is located 6-nt away from the ds/ss junction. Unwinding rates were not measured for substrates labeled n.a. (not applicable).

Given that YoaA was identified in a screen for factors that rescue stalled replication forks, different types of forked DNA structures were tested to further characterize the helicase activity. Unwinding of a DNA duplex containing either a 20-nt gap, a nick, or a nick with a 3ˈ flap (50 nM) (Table S3, substrates F6 - F8, see also Fig. 8) by YoaA-χ (50 nM) was measured using our FRET helicase assay. YoaA-χ unwound duplex DNA containing a 20-nt gap at a rate of 3.5 ± 0.2 × 10^−3^ s^−1^ (Fig. 7*A*, orange). This is similar to the rate of unwinding the overhang substrate (O1) in Figure 5 (Fig. 7*A*, black). YoaA-χ did not unwind duplex DNA with a nick nor nicked DNA with a 35-nt 3ˈ flap (Fig. 7*A*, green and blue, respectively).

To investigate the activity of YoaA-χ on a DNA duplex with a 5ˈ flap, a gel-based helicase assay was used instead of FRET to visualize the various types of unwound products. Unwinding of the DNA duplex with the 5ˈ flap structure would generate a 5ˈ ss overhang that YoaA-χ could translocate along to unwind the remaining duplex DNA. YoaA-χ (50 nM) successfully unwound the duplex with the 5ˈ flap from DNA containing either a 20-nt gap or nick (50 nM) (F9 and F10) at rates of 31 ± 3 × 10^−3^ s^−1^ and 25 ± 2 × 10^−3^ s^−1^, respectively (Fig. 7*B*). Interestingly, single-stranded Cy5-labeled template DNA was never observed in the gel helicase assay for either substrate, indicating YoaA-χ did not unwind the duplex with the 5ˈ ssDNA overhang that forms after removing the DNA with the flap (Fig. 7*B*, structures P2 or P5). This duplex was even refractory to complete denaturation by heat (Fig. 7*B*, lanes 4 and 13). To verify that the duplex with the 5ˈ overhang (structure P2) could not be unwound by YoaA-χ under these reaction conditions, an unwinding assay was done with this substrate only (data not shown). The difficulty to separate these strands either by heat denaturing or by YoaA-χ may be due to a G/C-rich sequence at the beginning of the duplex region at the ss/ds DNA junction.

### YoaA-χ unwinds damaged DNAs

Because *yoaA* and *holC* are critical genes for AZT tolerance in *E. coli* cells, we investigated the ability of YoaA-χ to unwind DNA terminated by AZT and other types of damage *in vitro* (16). Duplex DNA with a 35-nt 5ˈ ss overhang and AZT incorporated at the 3ˈ end of the primer (50 nM) (Table S3, substrate D1) was unwound by YoaA-χ (50 nM) at a rate of 1.8 ± 0.3 × 10^−3^ s^−1^ (Fig. 7*C*, purple). YoaA-χ (50 nM) also unwound 50 nM DNA with a 3ˈ dideoxy C at the 3ˈ end of the primer (D2) (3.8 ± 0.5 × 10^−3^ s^−1^) and DNA containing an abasic site on the translocating strand one nucleotide away from the ds/ss junction (D3) (1.9 ± 0.2 × 10^−3^ s^−1^) (Fig. 7*C*, yellow and light blue, respectively). Unwinding of the 3ˈ dideoxy C (3.8 ± 0.5 × 10^−3^ s^−1^) was slightly slower than the undamaged DNA substrate (O1) (4.7 ± 1 × 10^−3^ s^−1^) when unwound by the same preparation of YoaA-χ (Fig. 7*C*, yellow *versus* black). To determine if this was due to the missing 3ˈ OH on the sugar or because the damaged primer contained a 3ˈ C/G base pair instead of a T/A base pair, the rate of unwinding a duplex containing an undamaged C/G base pair (O3) was measured (Fig. 7*C*, pink). The rate of unwinding a substrate with a 3ˈ C/G base pair (3.1 ± 0.6 × 10^−3^ s^−1^) was also slower than the rate of unwinding the substrate with a 3ˈ T/A base pair but similar to the rate of unwinding the substrate with the 3ˈ dideoxy C (Fig. 7*C*). This indicates the slower rate of the 3ˈ dideoxy-C substrate is most likely due to the presence of the C/G base pair.

## Discussion

Human XPD/Rad3-like iron-sulfur helicases are critical enzymes involved in an array of genomic processes. They contribute to DNA replication and repair by resolving a variety of DNA structures, including D-loops, T-loops, and G-quadruplexes, that could hinder DNA metabolism (1). Mutations in XPD/Rad3-like helicases have been linked to genetic diseases and chromosomal abnormalities (12, 13, 14, 15). Less is known about the two *E. coli* XPD/Rad3-like helicase paralogs, DinG and YoaA, but it is becoming established that DinG and YoaA have roles in maintaining genomic integrity. Both DinG and YoaA help resolve various forms of DNA damage, but the degree to which they contribute to repairing specific lesions or resolving secondary nucleic acid structures varies and is not yet completely defined. The *yoaA* gene is necessary for cells to tolerate lesions that stall the replication fork, including AZT and MMS (16, 18). DinG contributes to the tolerance of AZT but to a lesser degree (16). Studies *in vivo* implicate DinG in helping resolve replication and transcription collisions and DinG unwinds R-loops *in vitro* (33, 34).

Helicases in XPD/Rad3 family are found throughout bacteria, but most only contain a single XPD/Rad3-like helicase. Gamma-Proteobacteria contain both YoaA and DinG, while other classes of bacteria in the phylum *Pseudomonadota*, including α, β, and δ-Proteobacteria, contain only one XPD/Rad3-like helicase that is more YoaA-like (16). Some Gram-positive bacteria contain a XPD/Rad3-like helicase that has gained an exonuclease domain and lost the Fe-S domain (16, 35). To verify that YoaA is a *bona fide* helicase and to better understand the differences between YoaA and DinG and why *E. coli* has two helicases, we have biochemically characterized the enzymatic activities of YoaA when YoaA is complexed with χ.

Foremost, we showed that YoaA and χ bind to form a stable YoaA–χ complex. This agrees with previous studies showing YoaA and χ contribute to resolving stalled forks and that YoaA binds a χ protein that is not a part of the DNA polymerase III holoenzyme (16, 19). The presence of a stable YoaA–χ complex is evident by the complex being present after three chromatographic steps (Ni^2+^ affinity, heparin, and gel filtration) and various salt conditions, ranging from 250 mM to 500 mM NaCl. We believe the physiologically relevant form of the helicase is YoaA in complex with χ. In the absence of χ, YoaA is mostly insoluble and has very weak ATPase and helicase activity. Chi could be needed for YoaA to have ATPase/helicase activity, though given the insolubility of YoaA when expressed in the absence of χ, it is likely that χ serves a structural role to stabilize YoaA and maintain an active enzyme. Chi could stabilize the folded structure of YoaA or reduce aggregation of YoaA by binding a hydrophobic area of YoaA. The function of χ in the YoaA-χ helicase will be further investigated.

Formation of a YoaA–χ complex was demonstrated by using two different size-exclusion chromatography columns, and in both cases, χ coeluted with YoaA. Although the two columns, when calibrated with standards, yielded slightly different molecular weights for the YoaA–χ complex, both are consistent in size with a complex that contains a single YoaA protein. The χ protein is small (16.6 kDa) relative to His-YoaA (72.6 kDa) and so based on these measurements alone, we cannot definitively conclude that there is only one χ present in the complex. However, there is no apparent symmetry in the YoaA protein that would suggest two χ-binding sites and χ is not known to form homodimers, therefore the protein complex likely has a stoichiometry of 1:1 YoaA:χ. Most of the XPD/Rad3-like helicases are monomeric, but FANCJ can form dimers (36).

In Figure 8, we show a side-by-side comparison of all the substrates used in our experiments and report rates of unwinding by YoaA-χ measured under the same experimental conditions (50 nM YoaA-χ, 50 nM DNA, and 2 mM ATP). Substrates that were not unwound by YoaA-χ under these conditions are in gray boxes. Substrates used for binding experiments are also shown in Figure 8 in yellow boxes. YoaA-χ has the conserved characteristics of an XPD/Rad-3 like helicase, including 5ˈ to 3ˈ directionality on DNA and helicase activity dependent on ATP hydrolysis (29, 30, 37, 38). Because YoaA-χ cannot unwind a 3ˈ overhang substrate (Fig. 8, O2), YoaA-χ does not translocate 3ˈ to 5ˈ on DNA nor can unwind blunt ends of DNA in the 5ˈ to 3ˈ direction. YoaA-χ does not need an ss-end to load onto DNA, because duplex DNA with a 20-nt gap was successfully unwound (Fig. 8, F6). The helicase activity of YoaA-χ is dependent on 5ˈ ss length. YoaA-χ had barely detectable helicase activity on duplex DNA with a 10-nt 5ˈ ss overhang (Fig. 8, F2), but the rate of unwinding greatly increased as the 5ˈ ss length increased up to 35-nt (Fig. 8, F1-F4). The helicase activity of YoaA-χ being dependent on 5ˈ ss length could be for several reasons. As 5ˈ ss length increases, so does the number of binding sites for YoaA-χ and therefore, the opportunities for YoaA-χ to bind the DNA. As 5ˈ ss length increases, multiple YoaA-χ molecules can bind the DNA at one time and prevent YoaA-χ from backsliding or prevent DNA from reannealing if one YoaA-χ falls off. Increasing 5ˈ ss length may also increase the processivity of YoaA-χ, as reported for other types of helicases (39, 40). Further mechanistic work will be done to determine how increasing the length of the ssDNA overhang stimulates YoaA-χ activity.

YoaA-χ unwinds bifurcated DNA/DNA substrates faster than substrates with only a 5ˈ ss overhang (Fig. 8, F1 *versus* O1), which is also the case for other XPD helicases, such as human FANCJ and CHLR1, and *E. coli* DinG (32, 34, 41). To determine whether this could be due to a difference in binding the two DNA substrates, binding of YoaA-χ to each was measured. The binding affinity of YoaA-χ for these DNA substrates is relatively weak, on the order of 300 to 400 nM, but similar in magnitude (Fig. 8, B2 and B3). Given that binding curves came close but did not reach the saturation point, it is possible that there is a 2 to 3 fold difference in *K*_*d*_ values that accounts for the rate difference. Introducing one unpaired nucleotide at the 3ˈ end of ds DNA did not increase the rate of unwinding by YoaA-χ to the same level as DNA with a 10-nt 3ˈ ss overhang (Fig. 8, F5 *versus* F1). This could mean the breaking of the first base pair by YoaA-χ is not the rate limiting step or that a longer displaced strand somehow stimulates YoaA-χ activity. More mechanistic helicase studies need to be conducted to determine how the displaced 3ˈ strand increases the rate of YoaA-χ unwinding.

Because YoaA-χ is implicated in resolving damage that stalls replication *in vivo*, we investigated the helicase activity of YoaA-χ on damaged DNA and forked DNA structures *in vitro.* Specifically, YoaA-χ was able to unwind DNA with AZT incorporated at the 3ˈ end of the primer, albeit slower than undamaged DNA (Fig. 8, D1 *versus* O1). It is known that *yoaA* and *holC* are necessary to resolve AZT-damaged DNA, but we also investigated the helicase activity of YoaA-χ on other types of damaged DNA. Substrates with a 3ˈ dideoxy C incorporated at the 3ˈ end of the primer or an abasic site on the translocating strand were both unwound by the helicase complex, with an abasic site having a slower rate than undamaged DNA (Fig. 8, D2 and D3). Archaeal XPD can also translocate across abasic sites, though only abasic sites within the duplex DNA were tested (42). YoaA-χ also unwound DNA structures that can arise from damage that halts replication, such as ssDNA gaps (Fig. 8, F6). YoaA-χ unwound duplex DNA, translocating along the lagging strand (5ˈ flap), (Fig. 8, F9 and F10), but could not unwind DNA with a 3ˈ flap (Fig. 8, F8), which is also the case for DinG and FANCJ (32, 34). The 5ˈ flaps with a 20-nt gap or nick were the two substrates YoaA-χ unwound the fastest, approximately twice the rate of unwinding a forked substrate (Fig. 8, F9 and F10 *versus* F1). All three substrates have a 35-nt 5ˈ ssDNA arm on which YoaA-χ translocates, but the flap substrates have a longer arm on the other side of the fork (F9, 40-nt or F10, 20-nt) than the forked substrate (F1, 10-nt). There could be a key binding site on YoaA-χ, similar to residue Y130 in *Tac* XPD, that interacts with the displaced strand and increases helicase activity (43). The 10-nt 3ˈ overhang may not be long enough to effectively bind such a site.

**Figure 9:**
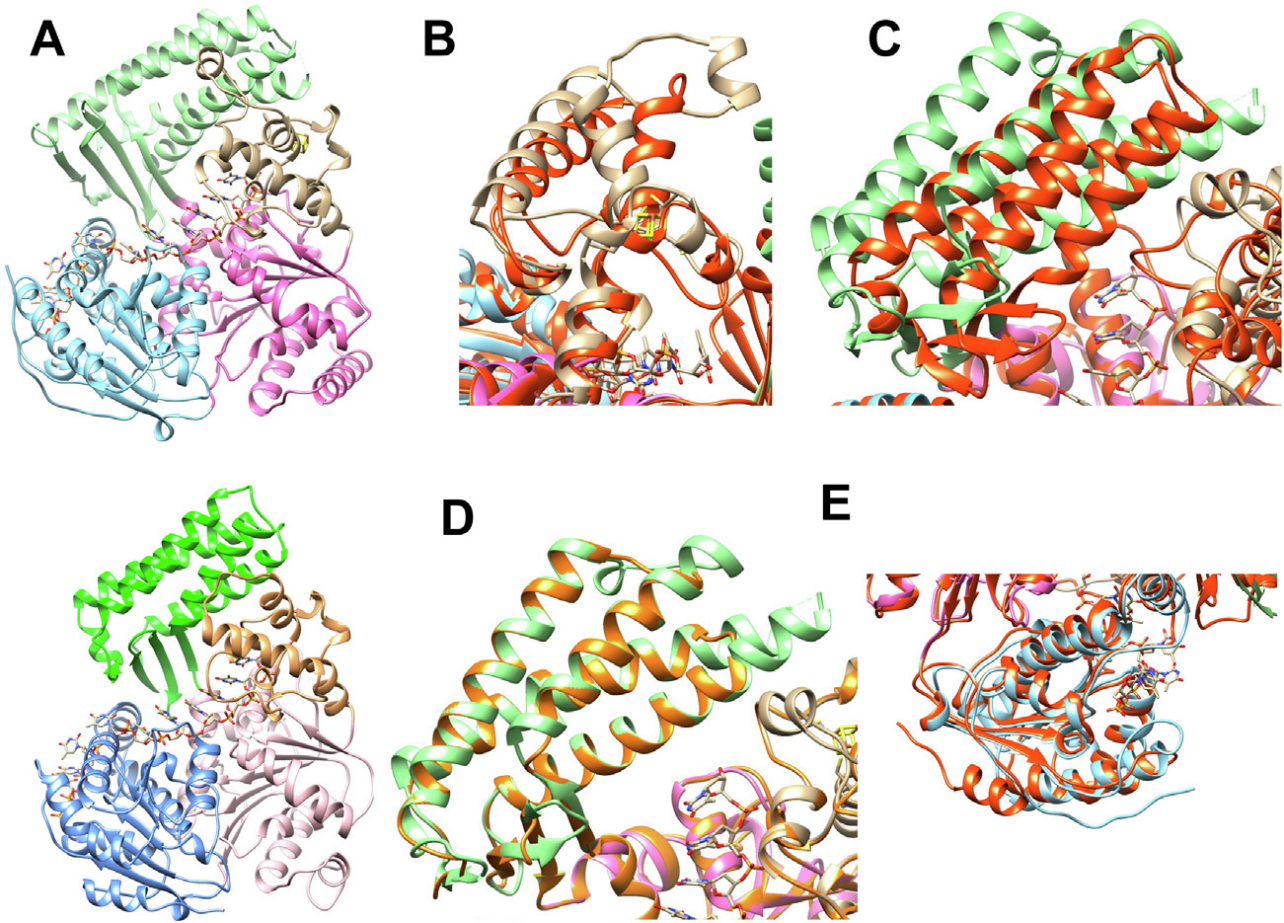
**Comparison of DinG and YoaA structures.***A*, crystal structure of DinG (PDB 6FWR) is shown in the *top* panel (44) and the predicted AlphaFold model of YoaA in the *bottom* panel (45, 46). Helicase domain I, helicase domain II, arch domain, and Fe-S domain are denoted in *pink*, *blue*, *green*, and *brown*, respectively. ssDNA is depicted in *stick* form. *B*, Fe-S domain of DinG (*brown*) and AlphaFold model of YoaA (*orange*) superimposed. *C*, arch domain of DinG (*green*) and AlphaFold model of YoaA (*orange*) superimposed. *D*, arch domain of DinG (*green*) and Raptor X model of YoaA (*light orange*) superimposed (47). *E*, C-terminal domain of DinG (*blue*) and AlphaFold model of YoaA (*orange*) superimposed.

DinG and YoaA share 29% amino acid sequence identity, and DinG is 80 amino acids larger than YoaA. DinG has a footprint size of 11-nt on ssDNA and we believe the footprint size of YoaA-χ on DNA is similar because YoaA-χ unwound DNA with a 20-nt gap (Fig. 8, F6) but had very little activity on DNA with a 10-nt 5ˈ ss overhang (Fig. 8, F2) (44). Amino acid sequence alignment of YoaA and DinG shows that DinG has insertions mainly in the arch and Fe-S domains that YoaA lacks (22). To determine how the extra 80 residues on DinG could lead to structural differences between DinG and YoaA, the AlphaFold model of YoaA was compared to the crystal structure of DinG (PDB 6FWR) (Fig. 9) (44, 45, 46). The model of YoaA shows high structural similarity to DinG within all four domains (Fig. 9*A*). Several additions in the crystal structure of DinG are present compared to the model of YoaA. One predicted structural difference is in the Fe-S domain, where DinG contains an extra alpha helix (Fig. 9*B*). This agrees with sequence alignment that shows DinG with extra insertions in the Fe-S domain. This predicted difference is also seen when DinG is overlaid with a Raptor X model of YoaA that was generated with DinG as the reference (Fig. S5). There is also a predicted structural difference in the arch domain. The AlphaFold model of YoaA predicts that the alpha helices in the arch domain of YoaA are slightly shorter and at a different angle, making the arch domain more closed than DinG (Fig. 9*C*). The alpha helices in the arch domain of the Raptor X model of YoaA are at the same angle as in DinG but they are shorter than DinG (Fig. 9*D*). These proposed structural differences between YoaA and DinG in the arch and Fe-S domains could confer substrate specificity for these helicases. The AlphaFold model of YoaA also predicts an alpha helix at the C-terminus but the Raptor X model of YoaA does not (Figs. 9*E* and S5). DinG does not contain an alpha helix at the C-terminus but the last 12 residues of DinG are not visible in the crystal structure so it is not known how these residues would fold (Fig. 9*E*). Genetic analysis indicate χ binds the C-terminus of YoaA (22). It is not likely that the extra 80 amino acids on DinG give rise to a χ-like function because these additional residues are mainly in the arch and Fe-S domains, not where χ is proposed to bind YoaA. Solving the structure of YoaA-χ, as well as in-depth studies on the types of structures YoaA-χ unwinds, will help reveal functional differences between these two helicases.

**Figure 10:**
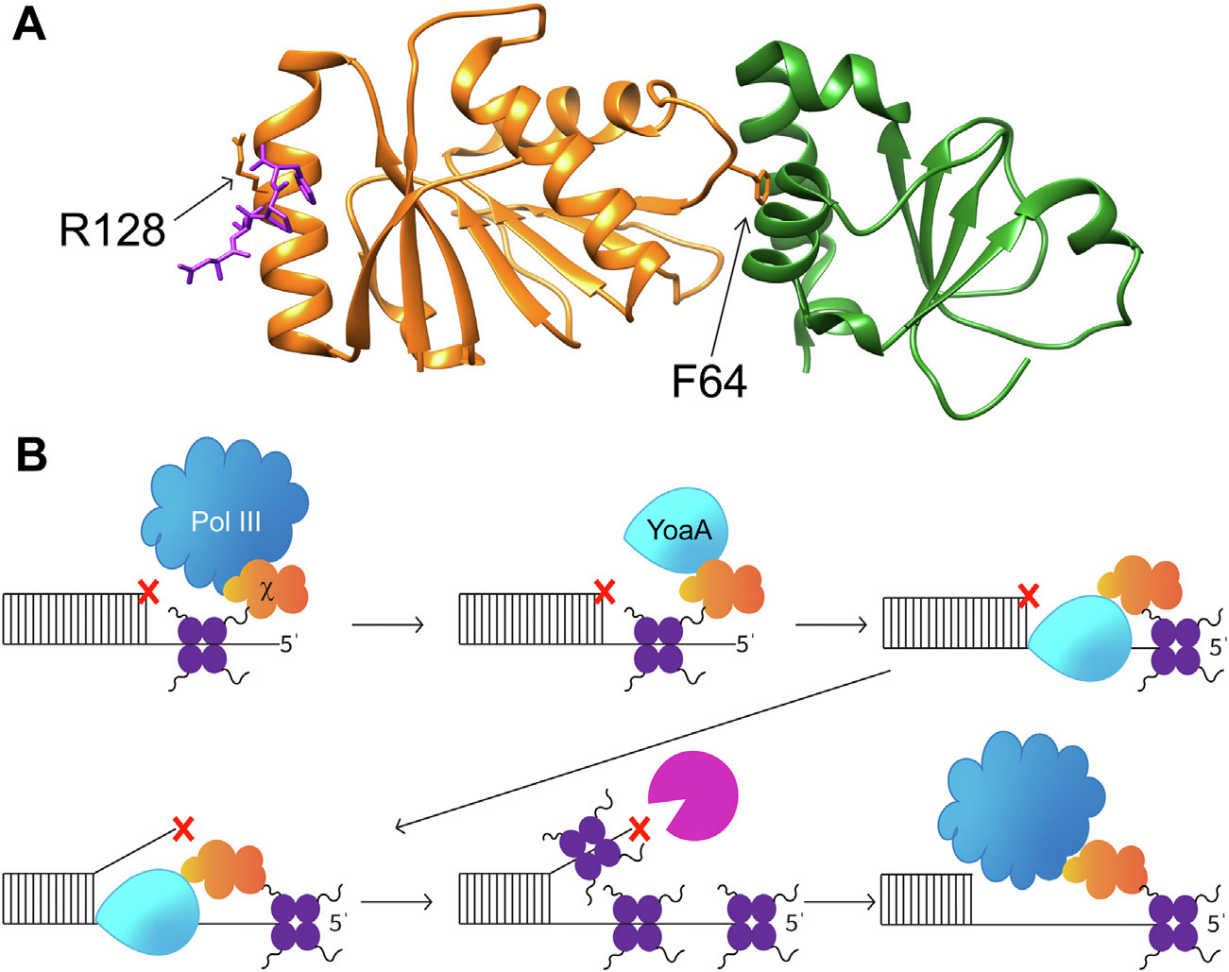
**Proposed model of YoaA-χ resolving stalled DNA replication.***A*, crystal structure of χψ bound to SSB with χ in *orange*, ψ in *green*, and SSB C-terminal peptide in *purple* in stick form (PDB 3SXU) (56). Residue F64 (*stick form*) on χ is important for interactions with YoaA and ψ. Chi residue R128 (*stick form*) is important for interactions with SSB. *B*, SSB (*purple*) coats ssDNA and the C-terminal tail of SSB interacts with χ (*orange*). Pol III HE (*dark blue*)-χ interacts with SSB on the lagging strand while replicating DNA. When pol III HE inserts a damaged nucleotide (*red* X) that terminates primer extension, replication stalls and ss-gaps can form. Chi-SSB interactions help recruit YoaA (*light blue*) to the stalled forked. YoaA-χ unwinds enough base pairs to allow for an exonuclease (*magenta*) to remove the DNA lesion. Pol III HE continues replication. SSB, ssDNA-binding protein.

The presence of χ with YoaA could be an evolutionary divergence between DinG and YoaA that maintains a key protein–protein interaction for the XPD/Rad3-like helicases with ssDNA-binding protein (SSB). SSB is an essential protein found in all domains of life that coats ssDNA and physically interacts with over a dozen DNA repair and replication proteins to coordinate their activities, including XPD/Rad-3 like helicases (reviewed in (43, 48)). The human homolog of SSB, replication protein A, interacts with and stimulates the activity of the human XPD/Rad-3 like helicase, FANCJ, and the *Fac* XPD is stimulated by *Fac* RPA2 (49, 50, 51, 52). The C-terminal acidic tip of *E. coli* SSB binds a hydrophobic pocket on the C-terminal end of χ and this SSB–χ interaction mediates SSB interactions with the pol III HE (53, 54, 55, 56, 57). We expect the SSB–χ interaction to be maintained in the YoaA–χ complex because YoaA binds to the N-terminal side of χ at or near Phe-64 whereas SSB binds the opposite side of χ near Arg-128 (19, 56) (Fig. 10*A*). Evidence suggests the SSB–χ interaction is necessary for YoaA-χ to repair damage that blocks replication because χ mutants that weaken SSB binding cannot promote AZT tolerance (16). The C-terminal acidic tip of SSB also interacts with DinG, but the SSB-binding site on DinG is not known (58). This SSB interaction stimulates the helicase activity of DinG (58). Because SSB binds DinG directly, DinG does not need χ to interact with SSB. We propose that the presence of χ with YoaA allows YoaA to maintain a similar interaction with SSB, and SSB coordinates YoaA-χ activity during DNA damage tolerance and repair.

A proposed model of how YoaA-χ works with DNA polymerase III and SSB to resolve damage that can stall the replication fork, such as AZT, is shown in Figure 10*B*. This model illustrates the two, distinct complexes χ forms at a stalled replication fork, one with the replicating polymerase, pol III HE, and another with YoaA to form an active helicase. In our proposed model, SSB binds ssDNA that forms during replication and that accumulates when forks are blocked by damage. Since one χ binds to one C-terminal tail of SSB, an SSB tetramer can potentially bind four χ′s (56). Pol III HE-χ interacts with SSB and pol III HE inserts a damaged base (red X) that stalls replication and causes ss-gaps to form. SSB coats the ss-gap, and the SSB–χ interaction helps recruit YoaA to the gapped DNA. Pol III HE dissociates from the damaged site or YoaA-χ−SSB interactions may destabilize the pol III HE–χ–SSB interactions to displace pol III HE to gain YoaA access to the DNA. In either case, YoaA-χ unwinds enough nascent DNA strand for an exonuclease to remove the damaged base from the 3ˈ end and DNA replication continues with pol III HE. We observed unwinding of damaged DNA by YoaA-χ was slower than undamaged DNA (Fig. 8), but perhaps the rate of unwinding damaged DNA will increase in the presence of protein-binding partners, such as SSB. Future studies will be focused on testing this model and determining how the SSB–χ interactions may affect YoaA activity.

## Experimental procedures

### Plasmid cloning

The *yoaA* gene with an N-terminal 6× histidine tag was cut out of pET15b with NcoI and BamHI and inserted into the multiple cloning site 1 of pCOLADuet-1 between the NcoI and BamHI sites. Correct insertion into the multiple cloning site 1 was verified by DNA sequencing which also confirmed that there were no mutations in *yoaA*.

### Site-directed mutagenesis of χ F64A and YoaA K51A

The *holC* gene in pET15b at residue phenylalanine 64 was mutated to alanine and the *yoaA* gene in pCOLADuet at residue lysine 51 was mutated to alanine with a Q5 Site-Directed Mutagenesis Kit (New England BioLabs) using the manufacturer’s protocol and the primers in Table S1. DNA sequencing confirmed that the desired mutations were made, and no other mutations were present.

### Overexpression of YoaA, YoaA-χ, YoaA-χ F64A, and YoaA K51A-χ

BL21 (DE3) *E. coli* cells were transformed with pCOLADuet-his-YoaA for the expression of YoaA, pCOLADuet-his-YoaA, and pET15b-HolC for the coexpression of YoaA and χ, pCOLADuet-his-YoaA and pET15b-HolC-F64A for the coexpression of YoaA and χ F64A, and pCOLADuet-his-YoaA K51A and pET15b-HolC for the coexpression of YoaA K51A and χ. The cells were grown at 37° C, with shaking at 250 RPM, in Terrific Broth with kanamycin (50 μg/ml) and carbenicillin (100 μg/ml) until an OD_600_ of approximately 0.6 was reached. IPTG (1 mM) was added to the cells to induce protein expression along with iron supplements, iron (II) sulfate (0.1 mg/ml) and ammonium ferric citrate (0.1 mg/ml). The cells were grown for 4 h more at 25 °C with shaking at 250 RPM. The cells were pelleted by centrifugation at 6370 RCF for 30 min, media was removed, and the cell pellets stored at −80 °C.

### Purification of proteins

Cell pellets were resuspended in cell lysis buffer/low imidazole buffer (20 mM sodium phosphate pH 7.8, 40 mM imidazole, 500 mM NaCl, 10% glycerol, Sigma FAST Protease Inhibitor Cocktail Tablet EDTA-free, and 2 mM DTT) and loaded onto a HisTrap FF column (Cytiva) and washed with low imidazole buffer. Proteins were eluted with a linear gradient of 40 mM to 500 mM imidazole with YoaA-χ eluting at approximately 100 mM imidazole. Fractions from the HisTrap elution were dialyzed overnight in dialysis buffer A (25 mM Tris–HCl pH 7.8, 500 mM NaCl, 10% glycerol, and 2 mM DTT) and then dialyzed for 6 h in dialysis buffer B (25 mM Tris–HCl pH 7.8, 250 mM NaCl, 10% glycerol, and 2 mM DTT). The samples were loaded onto a HiTrap Heparin HP column (Cytiva) and washed with low salt buffer (25 mM Tris–HCl pH 7.8, 250 mM NaCl, 10% glycerol, and 2 mM DTT). Proteins were eluted with a linear gradient of 0.25 to 1 M NaCl with YoaA-χ eluting at approximately 375 mM NaCl. Fractions containing YoaA-χ were dialyzed overnight into storage buffer (25 mM Tris–HCl pH 7.8, 250 mM NaCl, 30% glycerol, and 2 mM DTT) and stored at −80 °C. Protein concentration was measured using a Bradford assay and measuring the absorbance of pure YoaA-χ at 595 nm. Four different preparations of purified YoaA-χ were used in these studies to confirm the reproducibility. Technical repeats refer to multiple experiments using the same purified preparation of YoaA-χ. Biological repeats refer to multiple experiments using different purified preparations of YoaA-χ.

### Iron determination

The QuantiChrom Iron Assay Kit was used according to manufacturer’s instructions to quantitatively measure the amount of iron in three different purified preparations of YoaA-χ. Iron was measured once after purification through a HisTrap FF column and HiTrap Heparin HP column and measured again after gel filtration. Iron averaged around one iron per YoaA molecule before SEC and ranged from two to six irons per YoaA molecule after SEC. The increase in number of iron molecules after gel filtration suggest a component of the buffer or protein preparation interfering with the assay and is removed by gel filtration. The majority of YoaA-χ samples after gel filtration, though, were too dilute to give an accurate iron measurement within the linear range.

### Gel filtration of YoaA-χ

YoaA-χ purified as described above was dialyzed in dialysis buffer C (25 mM Tris–HCl pH 7.8, 250 mM NaCl, 10% glycerol, and 2 mM DTT). The protein was then concentrated to 2 mg/ml and loaded onto a Superose 12 10/300 column at 0.8 % column volume or concentrated to 0.8 mg/ml and loaded onto a Superdex 200 Increase 3.2/300 column at 1% column volume. The columns were washed with SEC buffer (25 mM Tris–HCl pH 7.8, 250 mM NaCl, 10% glycerol, and 2 mM DTT). Fractions containing YoaA-χ were analyzed by SDS-PAGE prior to pooling and dialyzing overnight into storage buffer and stored at −80 °C.

Gel filtration calibration standards (Bio-Rad) (vitamin B12 1.35 kDa, equine myoglobin 17 kDa, chicken ovalbumin 44 kDa, bovine γ-globulin 158 kDa, and bovine thyroglobulin 670 kDa) were loaded onto the Superose 12 10/300 column at 0.8% column volume or Superdex 200 Increase 3.2/300 column at 1% column volume and eluted with SEC buffer. Elution volumes were determined for each protein standard. Blue Dextran 2000 (1 mg/ml) was loaded onto a Superose 12 10/300 column at 0.8% column volume and Superdex 200 Increase 3.2/300 column at 1% column volume and washed with SEC buffer to determine the void volume of the column. A standard line was generated graphing K_av_ *versus* log (molecular weight) using Equation 1.

(1) $$K_{av}=\frac{v_{e}-v_{o}}{v_{c}-v_{o}}$$

where v_e_ is elution volume, v_c_ is geometric column volume, and v_o_ column void volume. The standard line was used to calculate the theoretical molecular weight of YoaA-χ and χ.

### SDS-PAGE

SDS-PAGE analyses used 12% tris-glycine gels stained with Coomassie Brilliant Blue. Gels were imaged with a Biorad Gel Doc.

### ATPase-coupled assay

ATPase activity was measured using a coupled enzyme assay and measuring the decrease in NADH absorbance (59). Reactions were performed in assay buffer A (42.5 mM Tris–HCl pH 7.5, 100 mM NaCl, 5 mM MgCl_2_, 50 μg/ml BSA, and 1 mM DTT). Final concentrations of 10 units pyruvate kinase and 16 to 18 units lactose dehydrogenase, 1 μM 60-nt ssDNA (Table S2, substrate S1), 2 mM phospho(enol)pyruvate, 150 μM β NADH, 2 mM ATP, and 25 nM YoaA-χ were used unless otherwise stated. NADH absorbance was recorded over time at 340 nm wavelength with a Cary Bio3 UV/Vis spectrophotometer. Rate of ATP hydrolysis was calculated by Equation 2. For the DNA titration assays, ssDNA (S1) concentrations of 20 nM, 30 nM, 50 nM, 75 nM, 100 nM, 200 nM, 300 nM, 450 nM, 600 nM, 750 nM, and 1000 nM were used, and the data were fit to a Michaelis-Menten–like equation (Equation 3) to calculate a *K*_*0.5*_ for three technical repeats. For MgCl_2_ dependence assays, MgCl_2_ concentrations of 0 mM, 2.5 mM, 5 mM, 10 mM, or 20 mM were tested. The rate of ATP hydrolysis was used to calculate the specific activity of each YoaA-χ preparation and for four different preparations, the rate of ATP hydrolysis ranged from 18 to 48 μM/min in reactions with 1 μM ssDNA (S1).

(2) $$rate=\frac{-\Delta \left({Abs}_{340}\right)/\Delta time}{\left(cuvet\;pathlength\;in\;cm\right)\left(6.22\frac{{mM}^{-1}}{cm}\right)}$$

(3) $$y=\frac{V_{\max}x}{K_{0.5}+x}$$

### DNA annealing

Oligonucleotides, with and without fluorophores attached, were purchased and purified either by HPLC if labeled or PAGE if unlabeled by Integrated DNA Technology. ssDNA substrates were mixed at equal concentrations in 20 mM Tris–HCl pH 7.5 and 50 mM NaCl, heated to 80 °C for 5 min, and cooled to room temperature over at least 4 h to anneal. Table S2 contains the DNA sequences of DNA substrates that were annealed to make the substrates in Table S3 and Figure 8.

### FRET-based helicase assay

DNA/DNA unwinding by YoaA-χ was measured by monitoring the increase in fluorescence of Cy3 over time. Helicase reactions were performed at room temperature with assay buffer B (50 mM Tris–HCl pH 7.5, 125 mM NaCl, 10 mM MgCl_2_, 50 μg/ml BSA, and 2 mM DTT), 2 mM ATP, and 50 nM annealed DNA (Table S3). The Cy3 fluorescence of the DNA was measured with a Photon Technology International QuantaMaster 1 fluorimeter, with excitation at 550 nm, emission at 565 nm, and a bandwidth of 4 nm. Purified YoaA-χ (2 nM, 5 nM, 10 nM, 20 nM, or 50 nM unless otherwise noted) was added to DNA substrates and ATP in assay buffer, and Cy3 emission was measured as a function of time (I_obs_) until the signal plateaued. The Cy3 signal for ds Cy3/Cy5 DNA without YoaA-χ was measured and averaged to determine the intensity of ds DNA (I_ds_). The Cy3 signal for ssDNA, without YoaA-χ, was measured and averaged to determine the intensity of ssDNA (I_ss_). The fluorescent signals obtained in DNA-unwinding reactions were converted to the concentration of DNA unwound by YoaA-χ using Equation 4.

(4) $$DNA\;unwound={DNA}_{total}*\left(\frac{I_{obs}-I_{ds}}{I_{ss}-I_{ds}}\right)$$

Observed reaction rates for each YoaA-χ concentration was calculated by fitting reaction time courses to exponential decays (Equation 5) using KaleidaGraph Software.

(5) $$y=C\left(1-e^{-kt}\right)+b$$

YoaA-χ concentration *versus* observed rate of reaction calculated from Equation 5 were graphed for three biological repeats and fitted with a line.

### Gel helicase assay

Helicase activity was visualized using a 10% native gel (10% acrylamide:bis solution, 19:1). To confirm DNA unwinding was occurring during the FRET helicase assay, 50 nM Cy5-labeled annealed DNA (Table S3, G1), 2 mM ATP, and either 5 nM or 2 nM YoaA-χ were mixed at room temperature with assay buffer B. Samples were removed from reactions at 30 s, 1 min, 2.5 min, 5 min, 10 min, and 30 min and quenched with 3 μM unlabeled ssDNA (Table S2 and S32), 1.5% SDS, 15 mM EDTA, and 37.5% glycerol (final concentrations after the quench solution is added are given). To monitor the unwinding of the 5ˈ flap duplex DNA with a 20-nt gap or a nick (Table S3, F9 and F10), 50 nM Cy5-labeled annealed DNA, 2 mM ATP, and 50 nM YoaA-χ were mixed at room temperature with assay buffer B. Samples were removed from reactions at 15 s, 30 s, 1 min, 5 min, and 15 min and quenched with 1.5% SDS, 15 mM EDTA, and 37.5% glycerol (final concentrations after the quench solution is added are given). For a positive control, YoaA-χ was not added to the reaction mix and the sample was heated at 95 °C for 5 min and put directly on ice. YoaA-χ was not added to the reaction mix and the sample was not denatured for the negative control. Samples were loaded onto a 10% native gel and run at 16W in a cold room for 2.5 h. The gel was imaged with an Amersham Typhoon and quantified using ImageQuantTL. Three technical repeats were performed for the gel helicase assays.

### Fluorescein-labeled DNA-binding assays

To measure the quench of fluorescein caused by YoaA-χ, substrates with a 30-nt DNA duplex and a 35-nt 5ˈ ss overhang labeled with a fluorescein on the duplex either 4-, 7-, 11-, 16-, or 20-nt away from the ss/ds junction (Table S3, B1) were used. YoaA-χ (1 μM) was added to these DNA substrates (50 nM) individually in assay buffer B with 0.5 mM ATPγS. An emission spectrum was taken of the DNA with the assay components excluding YoaA-χ at 495 nm excitation, 505 to 625 nm emission, and a 2 nm bandwidth to obtain a DNA-only emission spectrum. After YoaA-χ was added to the reaction, a time-based scan was taken at 495 nm excitation and 525 nm emission. Once the intensity signal plateaued, an emission spectrum was taken at 495 nm excitation and 505 to 625 nm emission to obtain a protein emission spectrum. The protein emission spectrum was made relative by dividing the protein emission spectrum by the DNA-only emission spectrum at 516 nm for each specific trial. Additionally, these relative protein emission spectra were further made relative to a no protein dilution control which was set to a value of 1. The relative fluorescein intensity was graphed *versus* fluorescein distance from the ss/ds junction for three technical repeats.

Overhang duplex DNA (Table S3, B2) and forked duplex DNA (Table S3, B3) (50 nM) labeled with a fluorescein 6-nt away from the ss junction was titrated with YoaA-χ (0.1 nM – 2000 nM) in assay buffer B with 0.5 mM ATPγS to measure DNA binding by YoaA-χ. Emission spectra and time-based readings were taken the same as above. The relative fluorescein intensity was plotted *versus* YoaA-χ concentration on a log x-axis. The data points were fit with a quadratic equation (Equation 6) to calculate a *K*_*d*_ value using KaleidaGraph Software, assuming a 1:1 protein:DNA binding. D_0_ is the total DNA concentration and E_0_ is the total YoaA-χ concentration. S_free,_ the maximum relative intensity, and S_bound_, the minimum relative intensity, were also fit as adjustable parameters S_free_ and S_bound_.

(6) $$y=\left(\left(\frac{D_{o}+E_{o}+K_{d}-\sqrt{{\left(D_{o}+E_{o}+K_{d}\right)}^{2}-4D_{o}E_{o}}}{2D_{o}}\right)*\left(S_{bound}-S_{free}\right)\right)+S_{free}$$

Experiments were performed with three technical repeats and graphed showing the average of three repeats and the SD.

### Polymerase incorporation of AZT into DNA

3ˈ-Azido-2ˈ,3ˈ-dideoxythymidine-5ˈ-triphosphate was purchased from Jena Biosciences. AZT was added to a 3ˈprimer end enzymatically in a reaction containing 500 μM AZT-TP, 10 μM primed template DNA (Table S3, substrate A1), 0.2 units/μl exonuclease-deficient Klenow fragment (New England Biolabs), and 1× Klenow assay buffer at 37 °C. The polymerase reaction was quenched after 45 min with 5 mM EDTA. A 95% formamide and 5% 0.5 M EDTA solution (twice the reaction volume) was added, and the sample was heat denatured at 95 °C for 5 min and put on ice. The AZT-strand was separated from unreacted primer and the template on a 12% denaturing acrylamide gel. The AZT-terminated primer was excised from the gel and extracted in 50 mM Tris base pH 7.5, 50 mM NaCl, and 1 mM EDTA and finally dialyzed against nanopure water. DNA concentration was measured *via* A_260_ and the AZT-terminated primer was annealed to substrate S2 (Table S2) using the above method.

### YoaA structural models

A structural model of YoaA was obtained from AlphaFold and a second model was predicted by Raptor X using *E. coli* DinG as a template (45, 46, 47).

## Data availability

Data that was not shown in this article can be shared upon request (lbloom@ufl.edu).

## Supporting information

This article contains supporting information (44).

## Conflicts of interest

The authors declare that they have no conflicts of interest with the contents of this article.
