# Supplementary material for: Characterization of the Escherichia coli XPD/Rad3 iron-sulfur helicase YoaA in complex with the DNA polymerase III clamp loader subunit chi (χ)
Source: J Biol Chem. 2022 Dec 9;299(1):102786. doi: 10.1016/j.jbc.2022.102786 (PMC9826845; doi:10.1016/j.jbc.2022.102786)
Supplement: Supplemental data [file mmc1.docx]

**Characterization of the *Escherichia coli* XPD/Rad3 iron-sulfur helicase YoaA in complex with the DNA polymerase III clamp loader subunit chi (χ)**

Savannah J. Weeks-Pollenz^1^, Yasmin Ali^1^, Leslie Morris^1^, Vincent A. Sutera^2^, Elizabeth E. Dudenhausen^1^, Margaret Hibnick^1^, Susan T. Lovett^2^, and Linda B. Bloom^1*^

**Supporting Information**

**Table S1: DNA primers to create mutants**

| **Primer Name** | **Sequence 5ˈ to 3ˈ** |
| --- | --- |
| χ F64A Forward Primer | GGCAGAAAGCGCGGTTCCGCATAATTTAGCGGGAGAAGGACC |
| χ F64A Reverse Primer | GGACGCGCCCACAGGGCT |
| YoaA K51A Forward Primer | CGGTACGGGCGCAACCTACGCT |
| YoaA K51A Reverse Primer | GTTCCTGCTTCCACCACC |

**Table S2: DNA Oligonucleotides**

| **DNA Substrate Name** | **DNA Length** | **Sequence (5ˈ to 3ˈ)** |
| --- | --- | --- |
| S1 | 60-nt | TTC AGG TCA GAA GGG TTC TAT CTC TGT TGG CCA GAA TGT CCC TTT TAT TAC TGG TCG TGT |
| S2 | 55-nt | TTT TTT TTT TTT TTT TTT TTT TTT TTT TTT TTT TTA TAA AAG GGA CAT TCT GGC C-Cy3 |
| S3 | 30-nt | Cy5-GGC CAG AAT GTC CCT TTT ATT ACT GGT CGT |
| S4 | 30-nt | GGC CAG AAT GTC CC**F** TTT ATT ACT GGT CGT |
| S5 | 55-nt | TTT TTT TTT TTT TTT TTT TTT TTT TTT TTT TTT TTA TAA AAG GGA CAT TCT GGC C |
| S6 | 20-nt | Cy5-GGC CAG AAT GTC CCT TTT AT |
| S7 | 55-nt | Cy3-CCG GTC TTA CAG GGA AAA TAT TTT TTT TTT TTT TTT TTT TTT TTT TTT TTT TTT T |
| S8 | 20-nt | TAT TTT CCC TGT AAG ACC GG-Cy5 |
| S9 | 85-nt | TTT TTT TTT TTT TTT TTT TTT TTT TTT TTT TTT TTT TTT TTT TTT TTT TTT TTT TTT TTT TTT TTA TAA AAG GGA CAT TCT GGC C |
| S10 | 30-nt | TTT TTT TTT TAT AAA AGG GAC ATT CTG GCC -Cy3 |
| S11 | 35-nt | TTT TTT TTT TTT TTT ATA AAA GGG ACA TTC TGG CC-Cy3 |
| S12 | 40-nt | TTT TTT TTT TTT TTT TTT TTA TAA AAG GGA CAT TCT GGC C-Cy3 |
| S13 | 21-nt | Cy5-GGC CAG AAT GTC CCT TTT ATT |
| S14 | 30-nt | GGC CAG AAT GTC CCT TTT ATT ACT GG**F** CGT |
| S15 | 30-nt | GGC CAG AAT GTC CCT TTT ATT AC**F** GGT CGT |
| S16 | 30-nt | GGC CAG AAT GTC CCT TTT A**F**T ACT GGT CGT |
| S17 | 30-nt | GGC CAG AAT G**F**C CCT TTT ATT ACT GGT CGT |
| S18 | 20-nt | GGC CAG AAT GTC CC**F** TTT AT |
| S19 | 20-nt | GTC CGC GAC TAA GGA TCA TT |
| S20 | 60-nt | AAT GAT CCT TAG TCG CGG ACT TTT TTT TTT TTT TTT TTT TAT AAA AGG GAC ATT CTG GCC-Cy3 |
| S21 | 40-nt | AAT GAT CCT TAG TCG CGG ACA TAA AAG GGA CAT TCT GGC C-Cy3 |
| S22 | 55-nt | Cy5-GGC CAG AAT GTC CCT TTT ATT ACT GGT CGT TTT TTT TTT TTT TTT TTT TTT TTT T |
| S23 | 55-nt | TTT TTT TTT TTT TTT TTT TTT TTT TTG CTG GTC ATT ATT TTC CCT GTA AGA CCG G |
| S24 | 20-nt | TTA CTA GGA ATC AGC GCC TG |
| S25 | 60-nt | Cy5-CCG GTC TTA CAG GGA AAA TAT TTT TTT TTT TTT TTT TTT TCA GGC GCT GAT TCC TAG TAA |
| S26 | 40-nt | Cy5-CCG GTC TTA CAG GGA AAA TAC AGG CGC TGA TTC CTA GTA A |
| S27 | 20-nt | Cy5-GGC CAG AAT GTC CCT TTT A**N** |
| S28 | 20-nt | Cy5-GGC CAG AAT GTC CCT TTT A**ddC** |
| S29 | 55-nt | TTT TTT TTT TTT TTT TTT TTT TTT TTT TTT TTT TTG TAA AAG GGA CAT TCT GGC C-Cy3 |
| S30 | 54-nt | TTT TTT TTT TTT TTT TTT TTT TTT TTT TTT TTT T**X**A TAA AAG GGA CAT TCT GGC C-Cy3 |
| S31 | 20-nt | Cy5-GGC CAG AAT GTC CCT TTT AC |
| S32 | 30-nt | GGC CAG AAT GTC CCT TTT ATT ACT GGT CGT |
| S33 | 19nt | Cy5-GGC CAG AAT GTC CCT TTT A |
| S34 | 65-nt | TTT TTT TTT TTT TTT TTT TTT TTT TTT TTT TTT TTA CGA CCA GTA ATA AAA GGG ACA TTC TGG CC |

Nucleotide (nt), fluorescein (F), AZT (N), 3ˈdideoxy-C(ddC), abasic site (X)

**Table S3: DNA substrates**

| **Experiment** | **DNA Substrate Name** | **Oligonucleotides annealed^1^** |
| --- | --- | --- |
| FRET helicase assay | F1 | S2 and S3 |
| Gel helicase assay | G1 | S3 and S9 |
| FRET helicase assay | F2 | S3 and S10 |
| FRET helicase assay | F3 | S3 and S11 |
| FRET helicase assay | F4 | S3 and S12 |
| FRET helicase assay | O1 | S2 and S6 |
| FRET helicase assay | O2 | S7 and S8 |
| FRET helicase assay | F5 | S2 and S13 |
| DNA binding assay | B1 | S4, S14, S15, S16, or S17 and S34 |
| DNA binding assay | B2 | S18 and S5 |
| DNA binding assay | B3 | S4 and S5 |
| FRET helicase assay | F6 | S6, S19, and S20 |
| FRET helicase assay | F7 | S6, S19, and S21 |
| FRET helicase assay | F8 | S19, S21, and S22 |
| Gel helicase assay | F9 | S23, S24, and S25 |
| Gel helicase assay | F10 | S23, S24, and S26 |
| FRET helicase assay | D1 | S2 and S27 |
| FRET helicase assay | D2 | S28 and S29 |
| FRET helicase assay | D3 | S6 and S30 |
| FRET helicase assay | O3 | S29 and S31 |
| AZT-incorporation assay | A1 | S5 and S33 |

^1^DNA sequences for substrates are in Table S2.

Fluorescence resonance energy transfer (FRET)

**
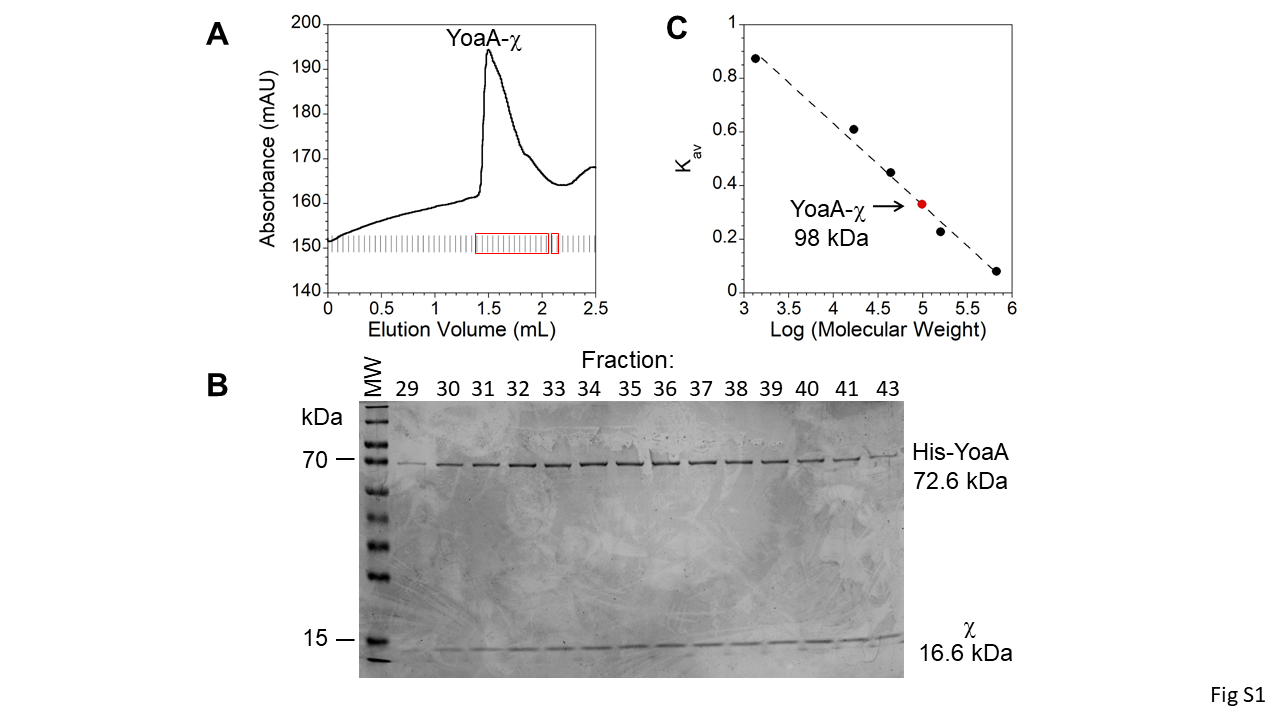
**

**Fig S1: Gel filtration of YoaA-χ by Superdex 200 Increase column. A.** The UV absorbance of column fractions eluted from a Superdex 200 Increase 3.2/300 gel filtration column is shown. The large peak corresponds to YoaA-χ. Vertical lines represent fractions and red box outlines fractions in panel **B**. **B.** SDS-PAGE analysis of fractions 29 through 41 and 43 eluted from SEC column is shown. MW denotes molecular weight ladder. Expected size of His-YoaA is 72.6 kDa and is 16.6 kDa for χ. **C.** The SEC column was calibrated with protein standards by graphing K_av_ versus log (molecular weight) (filled circles). The standard curve was fit to a line (R^2^ of 0.988) to calculate molecular weights of YoaA-χ (red filled circles). The size calculated for YoaA-χ on the Superdex 200 increase column is 98 kDa.


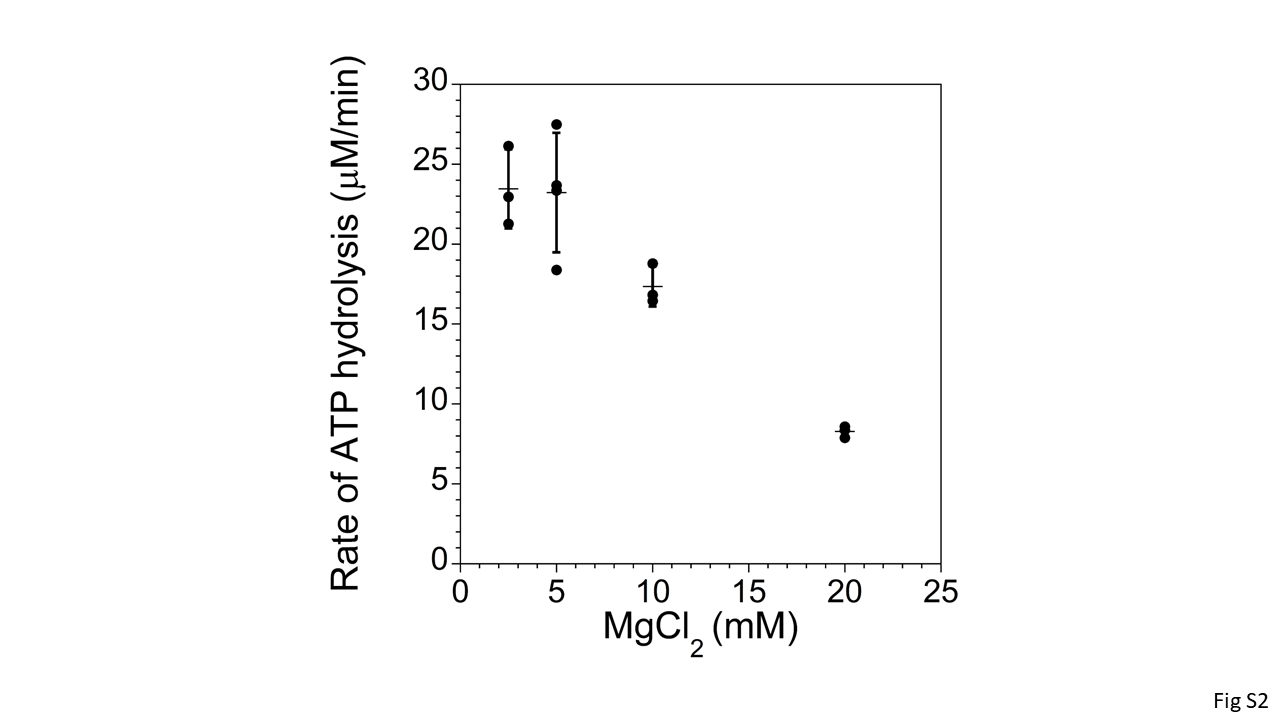


**Fig S2: ATPase activity of YoaA-χ is dependent MgCl_2_.** MgCl_2_ concentration ranged from 2.5 mM, 5 mM, 10 mM, and 20 mM with a 60-nt ss DNA (Table S2, S1) and 2 mM ATP. Filled circles represent three individual experiments, horizontal lines represent the average, and error bars represent standard deviations. There are four technical repeats for 5 mM MgCl_2_.


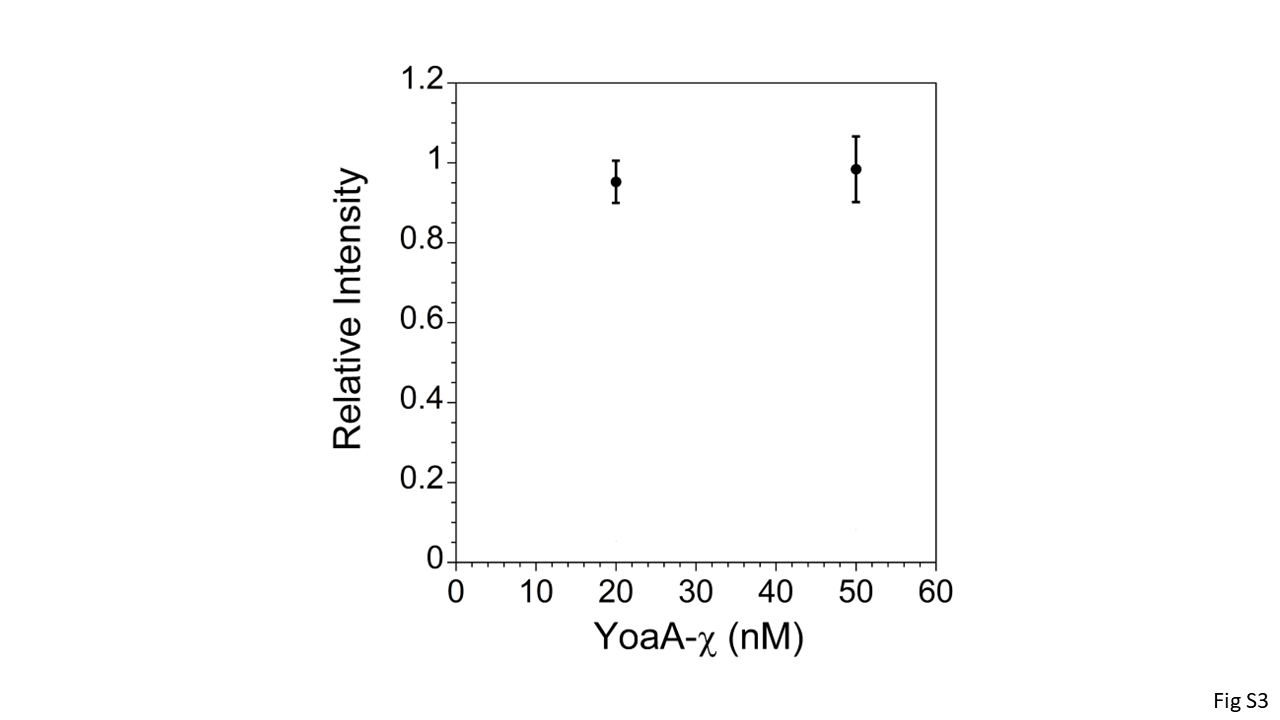


**Fig S3: Effect of YoaA-χ on Cy3 fluorescence in FRET helicase assay conditions.** Relative intensity of Cy3 substrate (50 nM, Table S2, substrate S2) at 567 nm when YoaA-χ is present at 20 nM and 50 nM. Experimental conditions match the conditions for the FRET helicase assay. Excitation at 540 nm, emission spectrum 550-650 nm, and 3 nm bandwidth. Filled circles represent average from three experiments and error bars represent standard deviations.


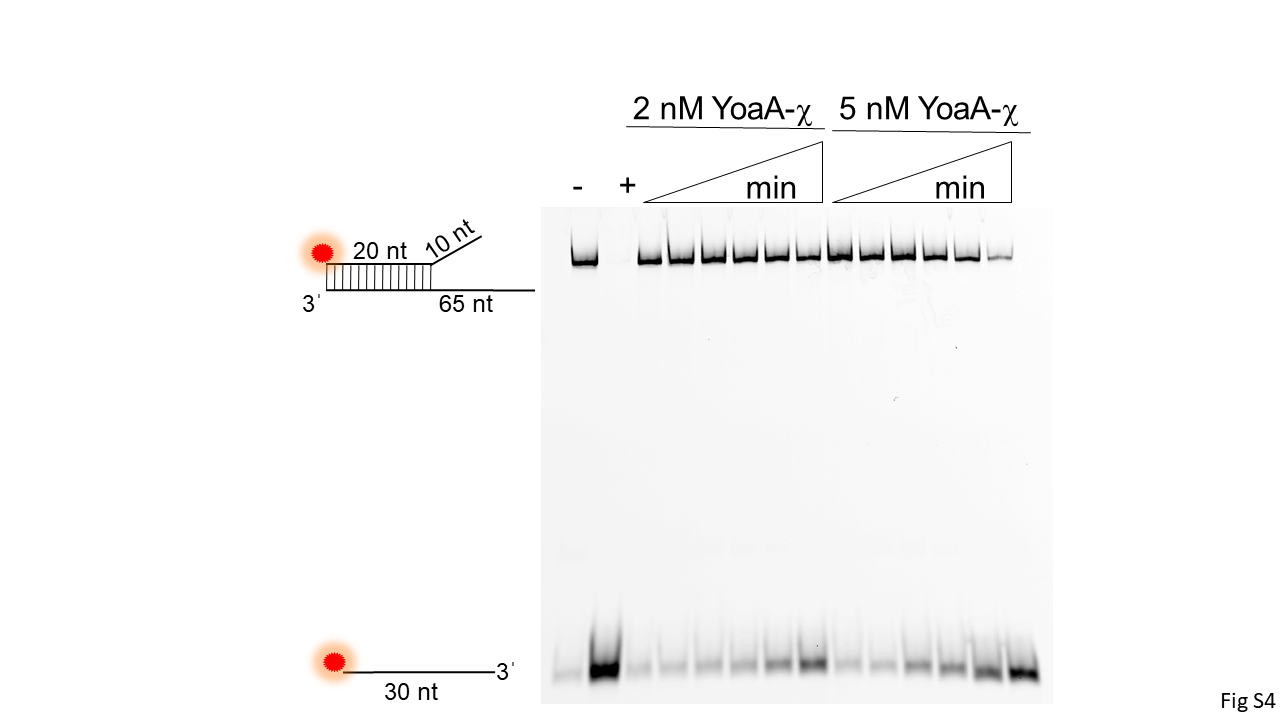


**Figure S4: DNA gel helicase assay of YoaA-χ shows DNA unwinding.** Native 10% acrylamide:bis gel showing DNA (50 nM, Table S3, G1) unwinding by either 2 nM or 5 nM YoaA-χ at 30 sec, 1 min, 2.5 min, 5 min, 10 min, and 30 min. The (+) lane is a positive control showing Cy5-labeled forked DNA duplex heated at 95°C for 5 min then directly placed on ice. The (–) lane is a negative control that contains no YoaA-χ and native DNA.


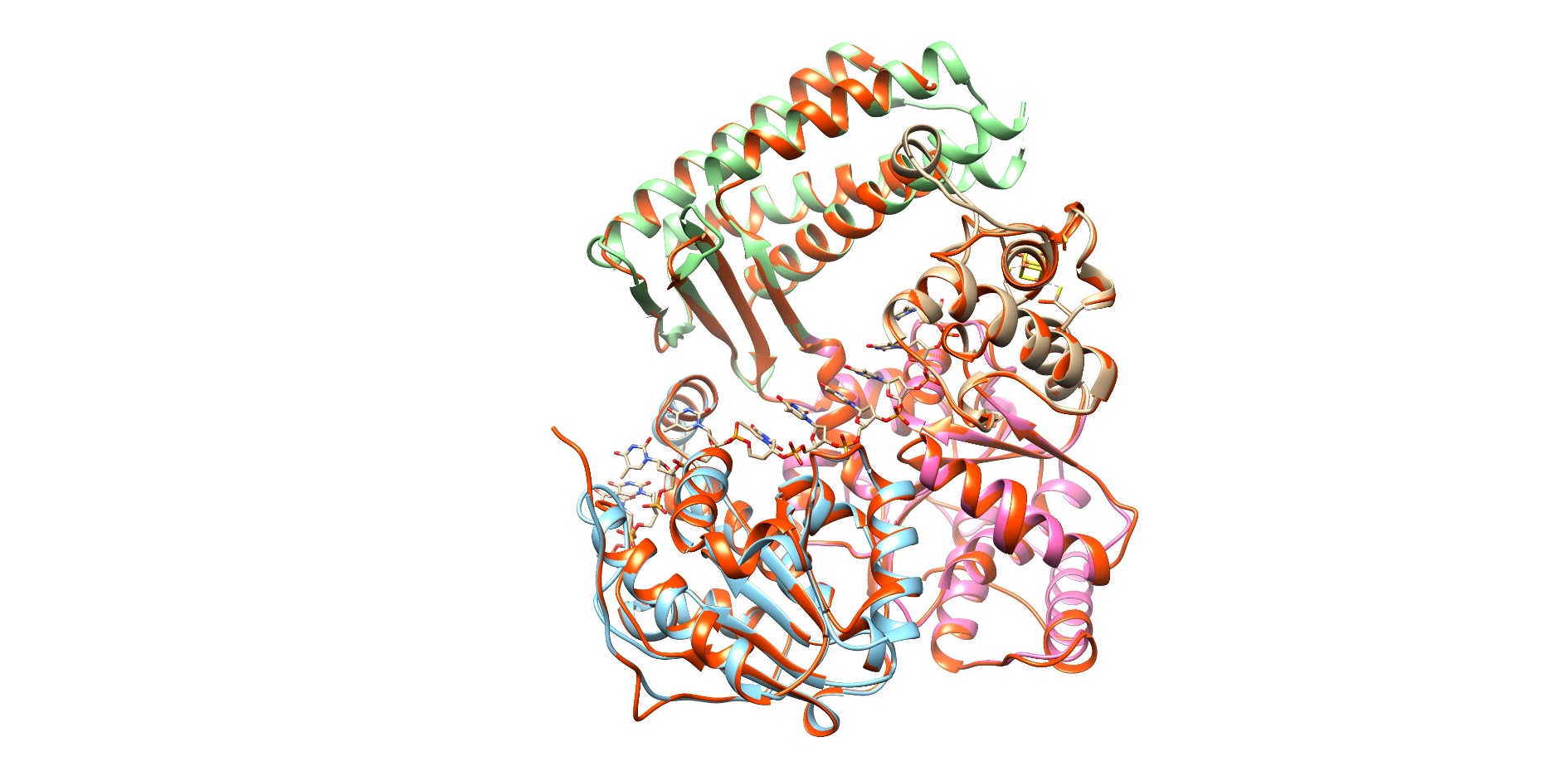


**Fig S5: Raptor X model of YoaA aligned with DinG.** Raptor X model of YoaA was generated using DinG as the reference structure. Model of YoaA (orange) is overlaid with crystal structure of DinG (PDB 6FWR) (40). Helicase domain I, helicase domain II, arch domain, and Fe-S domain of DinG are denoted in pink, blue, green, and brown respectively
